# Targeting SNRPE to Induce Pyroptosis Enhances Antitumor Immunity in Breast Cancer

**DOI:** 10.7150/ijms.109171

**Published:** 2025-04-28

**Authors:** Zaixiong Ji, Zilin Wang, Xinyu Guo, Junjian Li, Yiran Cai, Kangan Li

**Affiliations:** 1 Department of Radiology, Shanghai General Hospital, Shanghai Jiao Tong University School of Medicine, Shanghai 200080, P. R. China.; 2 Mini-invasive Interventional Therapy Center, Shanghai East Hospital, School of Medicine, Tongji University, Shanghai 200092, P.R. China.; 3 State Key Laboratory of Systems Medicine for Cancer, Department of Oncology, Shanghai General Hospital, Shanghai Jiao Tong University School of Medicine, Shanghai 200080, P. R. China.; 4 Department of Pathology, Shanghai General Hospital, Shanghai Jiao Tong University School of Medicine, Shanghai 200080, P. R. China.

**Keywords:** breast cancer, spliceosome, SNRPE, antitumor immunity, pyroptosis, prognosis

## Abstract

**Background:** Although SNRPE is a core spliceosomal component that guides pre-mRNA splicing in eukaryotic cells, its impact on mammary carcinoma prognosis and the immune microenvironment remains unclear. Pyroptosis, an inflammatory cell death, exerts tumor-suppressive functions and elicits antitumor immunity. Understanding the pathways that control pyroptosis will aid in developing specific antitumor strategies, while the relationship between SNRPE and pyroptosis has not been studied.

**Methods:** To determine the impact of SNRPE on tumor prognosis, survival analysis and immune infiltration assessment were performed on clinical samples from patients with breast cancer. The antitumor effects and further mechanisms of SNRPE targeting were investigated via the xenograft murine model and cell biology experiments.

**Results:** Here, we found that upregulation of SNRPE expression was associated with unfavorable tumor prognosis and low levels of immune infiltration. Our data identified SNRPE targeting activated natural killer (NK) cell-mediated antitumor immunity in breast cancer by triggering pyroptosis of tumor cells *in vivo*. SNRPE targeting modulated pyroptosis of tumor cells in a ROS-dependent manner.

**Conclusion:** This study contributes to new insights into the interaction between spliceosome-targeted tumors and host immunity, highlighting the targeting of spliceosome to trigger pyroptosis as a comprehensive therapeutic strategy for enhanced antitumor immunity in breast cancer.

## Introduction

Breast cancer has become the most prevalent newly diagnosed cancer type worldwide and remains the leading cause of cancer-related mortality^1^. Targeted therapies have been demonstrated to effectively improve the prognosis of breast cancer^2^, ^3^. By specifically inhibiting critical oncogenic pathways associated with cancer progression, targeted therapies exert its therapeutic effects^4^. Most studies in molecular targeted therapies have focused on kinases and the immune system^5^, ^6^. However, only a limited proportion of cancer cases respond favorably to these approaches, leaving the remaining patients subjected to potential ineffectiveness, side effects, and expensive treatments^7^. Therefore, it is necessary to explore alternative pathways associated with cancer progression. In recent years, targeting of spliceosome has been regarded as a promising avenue^8^.

The spliceosome is a macromolecular ribonucleoprotein complex that guides the splicing of precursor mRNA in eukaryotic cells^9^, ^10^. Small nuclear ribonucleoprotein polypeptide E (SNRPE) is a core spliceosomal component^11^, ^12^. Previous studies primarily focused on the impact of spliceosome inhibition on proliferation, migration, and the cell cycle of tumor cells^11^, ^13^. Recent research has indicated that splicing inhibition can trigger antiviral signaling in tumor cells^14^. Although studies on spliceosome inhibition to suppress certain cancers has been reported, the mechanisms underlying its selective tumor cell killing and its effects on the tumor microenvironment remain unclear. To the best of our knowledge, immune cell responses have been scarcely assessed in spliceosome-targeted tumors, and the natural killer (NK) cell response following spliceosome targeting in tumors has not yet been reported. Additionally, the potential effects of the immune response post spliceosome targeting in breast cancer on tumor activity have not been clarified.

Accumulating evidence emphasizes the significance of chronic inflammation in the pathological processes of breast cancer^15^. It is appealing to manipulate tumor immune microenvironment to impact its progression, treatment, and prognosis. In response to damage and pathogen-associated molecular patterns (PAMPs), the inflammasome is a protein signaling complex composed of the NLRP3 sensor, the apoptosis-associated speck-like protein (ASC, signal adapter), and caspase-1 protease^16^. Activated caspase-1 is essential for the release of pro-inflammatory cytokines Interleukin (IL)-1β and IL-18 and can cleave gasdermin D (GSDMD) to execute pyroptotic cell death^17^. Dysregulation of inflammasome activation can contribute to cancer development^18^. Interestingly, pyroptosis can influence the immune microenvironment of breast cancer^18^. Additionally, reactive oxygen species (ROS) serve as primary mediators in inducing NLRP3 inflammasome activation^19^. The imbalance in cellular redox homeostasis leading to ROS accumulation can trigger the assembly and activation of NLRP3 inflammasome and result in pyroptotic cell death^17^, ^19^. However, despite these findings, the role of pyroptosis in breast cancer has not been extensively studied.

**Scheme 1:**
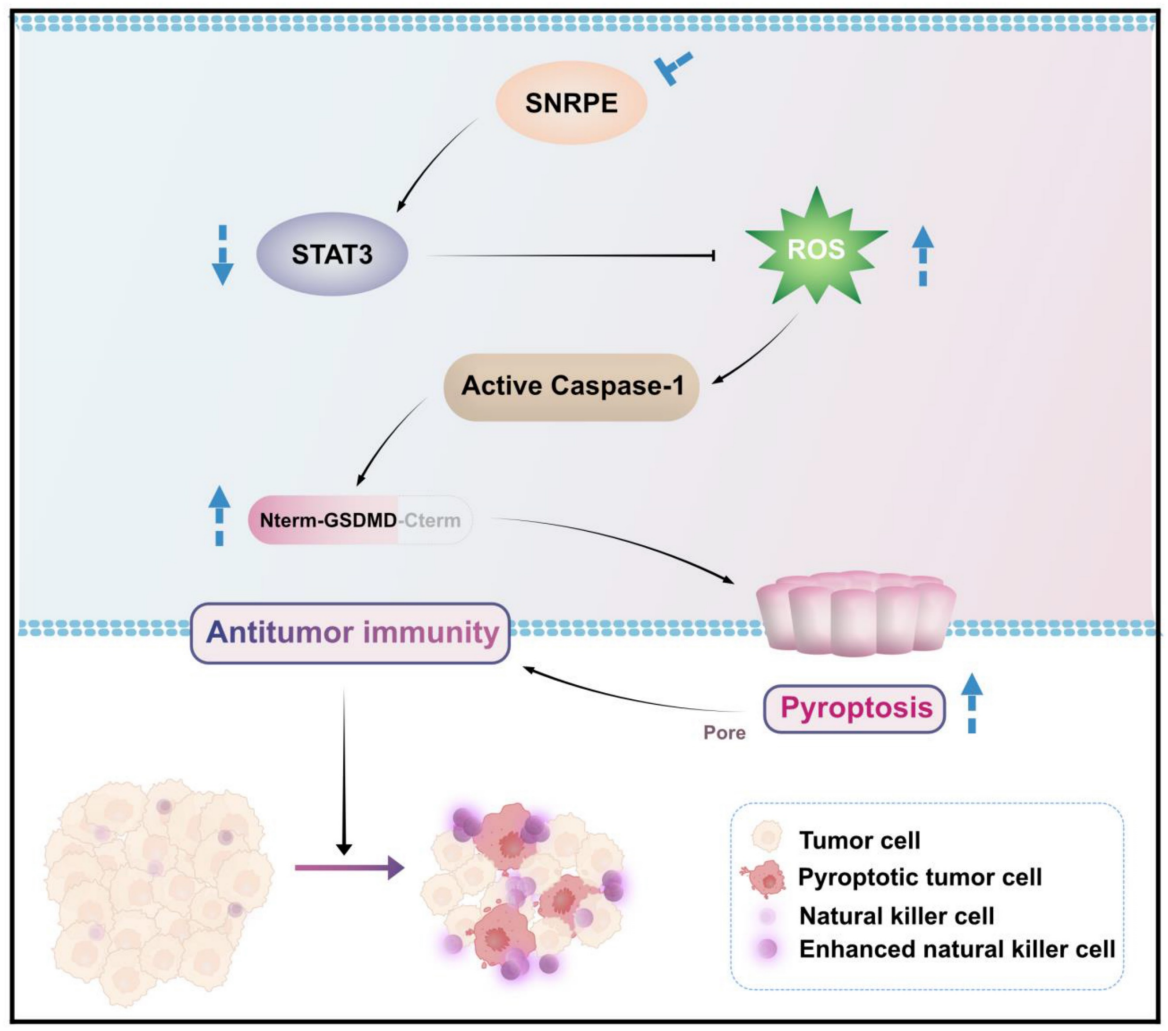
** Schematic illustration of how targeting dysregulated SNRPE orchestrates pyroptosis for enhanced antitumor immunity.** The depletion of SNRPE boosts ROS and triggers caspase-1/GSDMD processing as well as subsequent pyroptotic cell death in breast cancer cells, amplifying NK cell-mediated antitumor immunity.

In this study, we comprehensively analyzed the expression characteristics, prognostic value, and correlation with immune infiltration levels of SNRPE in breast cancer. Notably, SNRPE was more abundant in breast cancer and SNRPE expression correlated with the immune-suppressed microenvironment in clinical patients with breast cancer. Our data demonstrated that SNRPE targeting boosted reactive oxygen species (ROS), which triggered pyroptosis of tumor cells and thus amplified NK cell-mediated antitumor immunity in breast cancer *in vivo* (Scheme 1).

## Methods

### Cell culture and reagents

The human mammary carcinoma cells (MDA-MB-231, MCF7) and human non-malignant breast epithelial cells (MCF10A) were obtained from the National Collection of Authenticated Cell Cultures in Shanghai, China. MDA-MB-231 cells were cultured in Dulbecco's Modified Eagle's Medium (DMEM; GIBCO, USA) supplemented with 10% fetal bovine serum (FBS; GIBCO, USA) and 1% penicillin/streptomycin (P/S, GIBCO, USA). MCF10A cells were maintained in DMEM/F-12 (GIBCO, USA) supplemented with 5% horse serum (GIBCO, USA), 20 ng/ml epidermal growth factor (EGF; Sigma, USA), 10 μg/ml insulin, and 0.5 μg/ml hydrocortisone (Sigma, USA). MCF7 cells were cultured in MEM (GIBCO, USA) supplemented with 10% FBS (GIBCO, USA) and 1% P/S (GIBCO, USA). NK-92MI cells were maintained in MEMα (GIBCO, USA) supplemented with 0.2 mM inositol, 0.1 mM β-mercaptoethanol, 0.02 mM folic acid, 12.5% horse serum, 12.5% FBS, and 1% P/S. All cell lines were maintained in a dedicated incubator at 37°C with 5% CO_2_. When the cells reached a confluence of 70-80%, they were used for subsequent experiments. N-acetylcysteine (NAC) was purchased from Sigma Aldrich. CD49b rabbit antibody was purchased from Abclonal.

### RNA interference and lentiviral infection

SiRNA duplexes targeting SNRPE were designed and synthesized by RiboBio, China. The following human-specific siRNA was used: Human SNRPE siRNA (CCCTCGTGTTACTACAAGA). According to the manufacturer's instructions, the siRNA was transfected into cells at a final concentration of 50 nM using Lipofectamine 3000 reagent (Thermo Fisher, USA). After transfection, the transfection medium was replaced with regular growth medium after 8 hours. Lentiviral packaging was conducted with small hairpin RNA (shRNA) targeting mouse SNRPE, obtained from Yuan Biotech (Shanghai, China). MDA-MB-231 cells were infected with the lentiviral particles using a standard multiplicity of infection (MOI) of 10. MDA-MB-231 cells infected with shControl lentivirus were utilized as the control. Stable transduced cell lines were obtained by applying puromycin selection at a concentration of 5 μg/ml. For the knockdown of GSDMD, the sequence of shGSDMD was shGSDMD (GGAGACCATCTCCAAGGAACT).

### Tumor experiment *in vivo*

Four-week-old female BALB/c nude mice were obtained from the Experimental Animal Center of Shanghai General Hospital. All animal experiments were conducted in accordance with the principles of the Helsinki Declaration and approved by the Animal Ethics Committee of Shanghai General Hospital, Shanghai Jiao Tong University School of Medicine. At the beginning of the experiment, mice were subcutaneously injected with 2×10^6^ MDA-MB-231 cells, MDA-MB-231-Control cells, or MDA-MB-231-shSNRPE cells, designated as the Mock group, shControl group, and shSNRPE group, respectively (n=6 mice per group). The experiment lasted for 3 weeks, during which changes in tumor volume and body weight were recorded. Tumor volume (mm^3^) was calculated using the formula v=1/2×L×W^2^, where L represents the longest dimension, and W represents the perpendicular dimension. Three weeks after injection, all mice were euthanized by CO_2_ inhalation, and the tumor nodules were dissected and weighed.

### NK cell depletion *in vivo*

For NK cell depletion experiments, mice were treated weekly for three weeks with 35 μl of anti-Asialo-GM1 (ASGM1) antibody (Biolegend, USA) by intraperitoneal injection^20^.

### Immunoblot analysis

After cell lysis using a lysis buffer, the protein concentration was quantified using the BCA assay (Beyotime, China). Following protein separation on SDS-PAGE gel, they were transferred to a polyvinylidene difluoride (PVDF; Millipore, USA) membrane. After adequate incubation of the proteins on the PVDF membrane with their corresponding antibodies, the chemiluminescent detection system was used to detect the immunolabeled proteins. β-actin or β-tubulin was used as a loading control. The primary antibodies used were as follows: SNRPE (Abcam, UK), Cyclin D1 (Abcam, UK), PCNA (Abcam, UK), Cleaved caspase-1 (Cell Signaling Technology, USA), Cleaved GSDMD (Abcam, UK), STAT3 (Abcam, UK), β-actin (Cell Signaling Technology, USA), β-tubulin (Cell Signaling Technology, USA). HRP-conjugated goat anti-mouse IgG (Cell Signaling Technology, USA) and HRP-conjugated goat anti-rabbit IgG (Cell Signaling Technology, USA) were used as secondary antibodies. The experiments were performed with at least three independent replicates.

### Cell viability assay

The Cell Counting Kit-8 (CCK-8; Dojindo, Japan) assay kit was used for a relatively simple and accurate analysis of cell viability. In brief, cells were seeded in a 96-well plate and incubated in a 37°C CO_2_ incubator. Once the cell confluence reached 70-80%, the cells were further cultured for 24 hours in serum-free medium. Then, 10 μl of CCK-8 working solution was added to each well of the 96-well plate. After gentle mixing, the plate was placed back into the cell culture incubator for a 1-hour reaction. The optical density (OD) value was measured at a wavelength of 450 nm using a microplate reader.

### Mitochondrial membrane potential assay

The JC-1 probe (Beyotime, China) was used to detect mitochondrial membrane depolarization. Specifically, cells were seeded in a 6-well plate and allowed to adhere. After that, JC-1 working solution was added to the cells and incubated at 37°C for 20 minutes. The cells were then observed under a fluorescence microscope. The experiment was repeated three times.

### ROS detection

The cells were seeded in a 6-well plate and allowed to proliferate until they reached 80% confluency. After that, the medium was replaced with serum-free medium for further cultivation for 24 hours to eliminate interference from serum. A 1:1000 dilution of 7'-dichlorodihydrofluorescein diacetate (DCFH-DA) was prepared in serum-free medium. The cell culture medium was removed, and 1 ml of the diluted DCFH-DA-containing medium was added to each well of the 6-well plate. The plate was then incubated at 37°C in a cell culture incubator for 20 minutes. After incubation, the cells were washed three times with serum-free cell culture medium to thoroughly remove extracellular DCFH-DA. The fluorescence intensity of each well was detected and photographed using an excitation wavelength of 488 nm. For Dihydroethidium (DHE) staining, tumor tissues were immediately embedded in an optimum cutting temperature (OCT) compound, snap-frozen in ethanol-dry ice, and stored at -80°C. Unfixed frozen samples were sectioned into 5μm-thick slices and placed onto glass slides. Each tissue section was treated with 10 μM DHE working solution. The slides were incubated in a temperature-controlled chamber at 37°C for 30 minutes. Ethidium fluorescence (excitation at 490 nm, emission at 610 nm) was visualized using fluorescence microscopy.

### Immunofluorescence assay

To determine the expression level of the target protein, immunofluorescence assay was employed. Briefly, collected cells were fixed and permeabilized. Subsequently, they were incubated overnight at 4°C with anti-STAT3 antibody (1:200 dilution, Abcam, UK). After washing with PBS, the cells were incubated with Alexa Fluor 488-labeled goat anti-rabbit IgG (Abcam, UK) for 1 hour in the dark. DAPI staining was performed to visualize the cell nuclei. After 5 minutes, the samples were observed and photographed using a fluorescence microscope (DMI8, Leica, Germany). The fluorescence intensity was calculated as the average of at least six independent fields per group.

### Immunohistochemistry

The specific steps were as previously described^21^. Briefly, after dewaxing the paraffin-embedded tissue sections, antigen retrieval was performed using sodium citrate buffer. The sections were then incubated at 37°C with 3% hydrogen peroxide for 10 minutes, followed by blocking with 5% bovine serum albumin (BSA) for 30 minutes. After applying the primary antibody, the sections were incubated overnight at 4°C. Signal detection was performed using HRP-conjugated rabbit secondary antibody and SABC (Boster, China), and the sections were counterstained with hematoxylin. Image analysis was conducted using Image-Pro Plus 6.0 software.

### Live/dead cell detection

Mammary carcinoma cells transfected with siSNRPE or siControl RNAs were co-cultured separately with NK cells, followed by co-culturing the isolated NK cells separately with tumor cells. Calcein-AM and propidium iodide (Beyotime, China) were employed to stain live and dead cells, respectively, in tumor cell samples, enabling the evaluation of NK cell cytotoxicity against tumor cells.

### NK cells cytotoxicity assay *in vitro*

NK cells were isolated from spleen and tumor draining lymph nodes of mice by magnetic separation using the Easy Sep Mouse NK Cell Isolation Kit (Stemcell, Canada) according to the manufacturer's protocol. MDA-MB-231 mammary carcinoma cells were seeded in 96-well plates at a density of 5000 cells per well 24 h prior to co-culture. Isolated NK cells and tumor cells were co-cultured in complete RPMI medium supplemented with 5 ng/mL IL-2 (Peprotech, USA). After 24 h of co-culture, the cytotoxicity of NK cells was assessed using the LDH Cytotoxicity Assay Kit (Beyotime, China). Wells containing only target cells without the addition of NK cells were used as sample controls to ascertain spontaneous cell death during the assay. Tumor cells lysed with 2% Triton X-100 served as a fully lysed positive control.

### Survival prognosis analysis

Kaplan Meier Plotter is an online website used to evaluate the impact of 54,000 genes on the survival in 21 types of cancer^22^. We analyzed the correlation between SNRPE expression and survival in breast cancer. The hazard ratio (HR) with a 95% confidence interval and the log-rank *P*-value was calculated accordingly.

### Functional enrichment analysis of SNRPE

Use the cBioPortal dataset to identify genes that were co-expressed with SNRPE^23^. In addition, genes correlated to SNRPE were obtained through the "Similar Gene Detection" module of GEPIA2 (http://gepia2.cancer-pku.cn/#index) based on all TCGA tumors and normal tissue data sets^24^. Then, we used the Venn diagram online analysis website to conduct cross-analysis, and obtained the genes shared by the above two gene lists^25^. Further, using Gene Ontology (GO) and Kyoto Encyclopedia of Genes and Genomes (KEGG) analyzed the functions and pathways of the two sets of genes in Metascape^26^. The terms with P-value < 0.01, minimum count 3, and enrichment factor > 1.5 were collected through Metascape, and they were grouped according to the similarity of their members to realize data visualization. GO enrichment analysis predicted the function of these genes. KEGG analysis indicated the metabolic and regulatory pathways of SNRPE-related genes from a molecular network perspective.

### Genetic alteration analysis

cBioPortal was used for the genetic alteration analysis^23^. Specifically, after selecting "TCGA Pan Cancer Atlas Studies" in the "Quick Search" section, we entered "SNRPE" to look up the genetic alteration properties of SNRPE. In the "Cancer Types Summary" module, genetic alteration frequency, mutation type, and CNA (copy number alteration) were observed for all TCGA tumors.

### Immune infiltration analysis

TIMER is a web server for the comprehensive analysis of tumor-infiltrating immune cells^27^. The correlation between SNRPE expression and immune mediators including fibroblasts, B cells, CD4^+^ T cells, CD8^+^ T cells, neutrophils, macrophages, and dendritic cells was explored using its "immune gene" module in pan-cancer from TCGA. Then, we used the correlation module to study the correlation between the expression of SNRPE and the biomarkers of specific tumor-infiltrating immune cell subsets, and visualized the data as heat maps and scatter plots. The levels of immune infiltration were evaluated by TIMER, CIBERSORT, CIBERSORT-ABS, QUANTISEQ, XCELL, MCPCOUNTER, and EPIC algorithms. Estimated statistical significance and partial correlation (cor) values ​​were obtained by Spearman's rank correlation test with purity adjustment.

### Statistical analysis

Perform statistical analysis using GraphPad Prism 8 software. Data from at least three independent experiments were presented as mean ± standard deviation. Differences between multiple groups were assessed using one-way analysis of variance (ANOVA), and student's t-test was used to compare two groups. Spearman's correlation analysis was used to measure the degree of correlation between specific variables. The strength of the correlation was determined using the absolute values of the following r values: 0.00-0.19 "very weak", 0.20-0.39 "weak", 0.40-0.59 "moderate", 0.60-0.79 "strong", and 0.80-1.0 "very strong". *P*-value < 0.05 were regarded statistically significant.

## Results

### SNRPE defines poor prognosis and correlates with immune-suppressed microenvironment in patients with breast cancer

**Figure 1:**
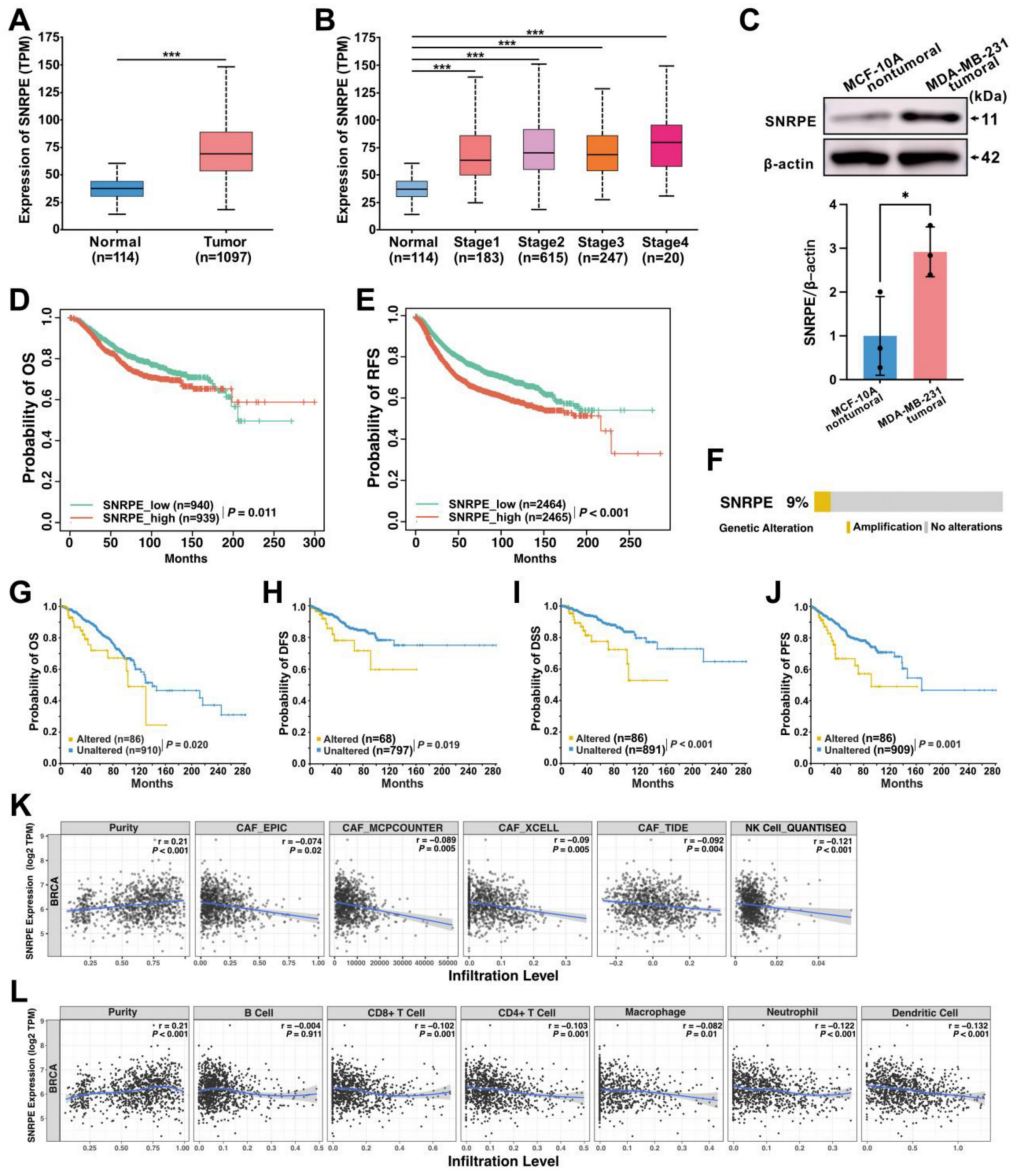
**SNRPE defines poor prognosis and correlates with immune-suppressed microenvironment in patients with breast cancer. A** Analysis of SNRPE mRNA levels in clinical samples of patients with breast cancer, procured from the TCGA database. **B** Levels of SNRPE mRNA in patients with breast cancer across various stages. **C** Evaluation of SNRPE protein expression levels in nontumoral and tumoral cells by western blots, with relative intensity normalized to β-actin (n=3). **D, E** Kaplan-Meier survival curves for overall survival (OS) and recurrence-free survival (RFS) in patients with breast cancer, stratified based on high or low expression levels of SNRPE. **F** Genetic alterations of SNRPE in patients with breast cancer (n=996). **G, H, I, J** Kaplan-Meier survival curves for overall survival (OS), disease-free survival (DFS), disease-specific survival (DSS), and progression-free survival (PFS) in patients with breast cancer, stratified according to the status of SNRPE alterations (altered vs unaltered). **K** Analysis of the correlation between SNRPE expression and cancer-associated fibroblast (CAF) immune infiltration levels in BRCA based on different algorithms (n=1100); analysis of the correlation between SNRPE expression and the infiltration level of NK cells in BRCA (n=1100). **L** Analysis of the correlation between SNRPE expression and the infiltration levels of macrophages, neutrophils, dendritic cells, B cells, CD8+ T cells, and CD4+ T cells in BRCA (n=1100). Data are expressed as means ± SD. *P < 0.05, ***P < 0.001.

To retrospectively evaluate the expression of SNRPE and its correlation with prognosis in patients with breast cancer, we analyzed clinical samples from 1,211 patients obtained from the TCGA database and discovered a significant increase in SNRPE mRNA levels in breast cancer tissues compared to adjacent normal tissues (Figure 1A). Next, we conducted an evaluation of SNRPE levels across different tumor stages. The results revealed a significant elevation of SNRPE levels in patients with breast cancer at stages 1-4, and notably, SNRPE levels were consistently higher in relatively poorer tumor stages (Figure 1B). Concurrently, we assessed the expression levels of SNRPE in normal human breast epithelial cells (MCF10A) and human mammary carcinoma cells (MDA-MB-231). Consistently, the expression level of SNRPE was significantly elevated in tumor cells compared to normal breast epithelial cells (Figure 1C). Similarly, the levels of SNRPE in MCF-7 mammary carcinoma cells also were increased (Figure S1A). To investigate the correlation between SNRPE and prognosis in breast cancer, we performed survival correlation analysis for overall survival (OS) and recurrence-free survival (RFS) (Figure 1DE). Remarkably, higher level of SNRPE was associated with shorter OS and RFS. These results indicate that SNRPE is overexpressed in breast cancer and defines a poor prognosis. Furthermore, we examined the genetic alterations of SNRPE in breast cancer and found that SNRPE exhibited only gene amplification in breast cancer, with an alteration frequency of 9% (Figure 1F). Notably, the altered group showed significant associations with shorter OS, DFS, DSS, and PFS compared to the unaltered group (Figure 1GHIJ). These data show that genomic amplification of SNRPE indicates an adverse prognosis in patients with breast cancer.

To explore the impact of SNRPE on the immune microenvironment in breast cancer, we retrieved RNA-seq data and corresponding clinical information from 1,100 patients with breast cancer in the TCGA database for performing immune infiltration analysis. Notably, based on algorithms including EPIC, MCPCOUNTER, XCELL, and TIDE, the expression level of SNRPE showed a significant negative correlation with the infiltration level of cancer-associated fibroblasts (CAFs) in BRCA (Figure 1K). Furthermore, SNRPE expression exhibited significant negative correlations with the infiltration levels of macrophages, neutrophils, dendritic cells, CD8^+^ T cells, and CD4^+^ T cells in BRCA (Figure 1L). Notably, the expression level of SNRPE also demonstrated a negative correlation with natural killer (NK) cell infiltration in BRCA (Figure 1K). These findings suggest a correlation between dysregulated overexpression of SNRPE and immune-suppressed microenvironment in breast cancer.

### Targeting dysregulated SNRPE in breast cancer exerts the antitumor effects

**Figure 2:**
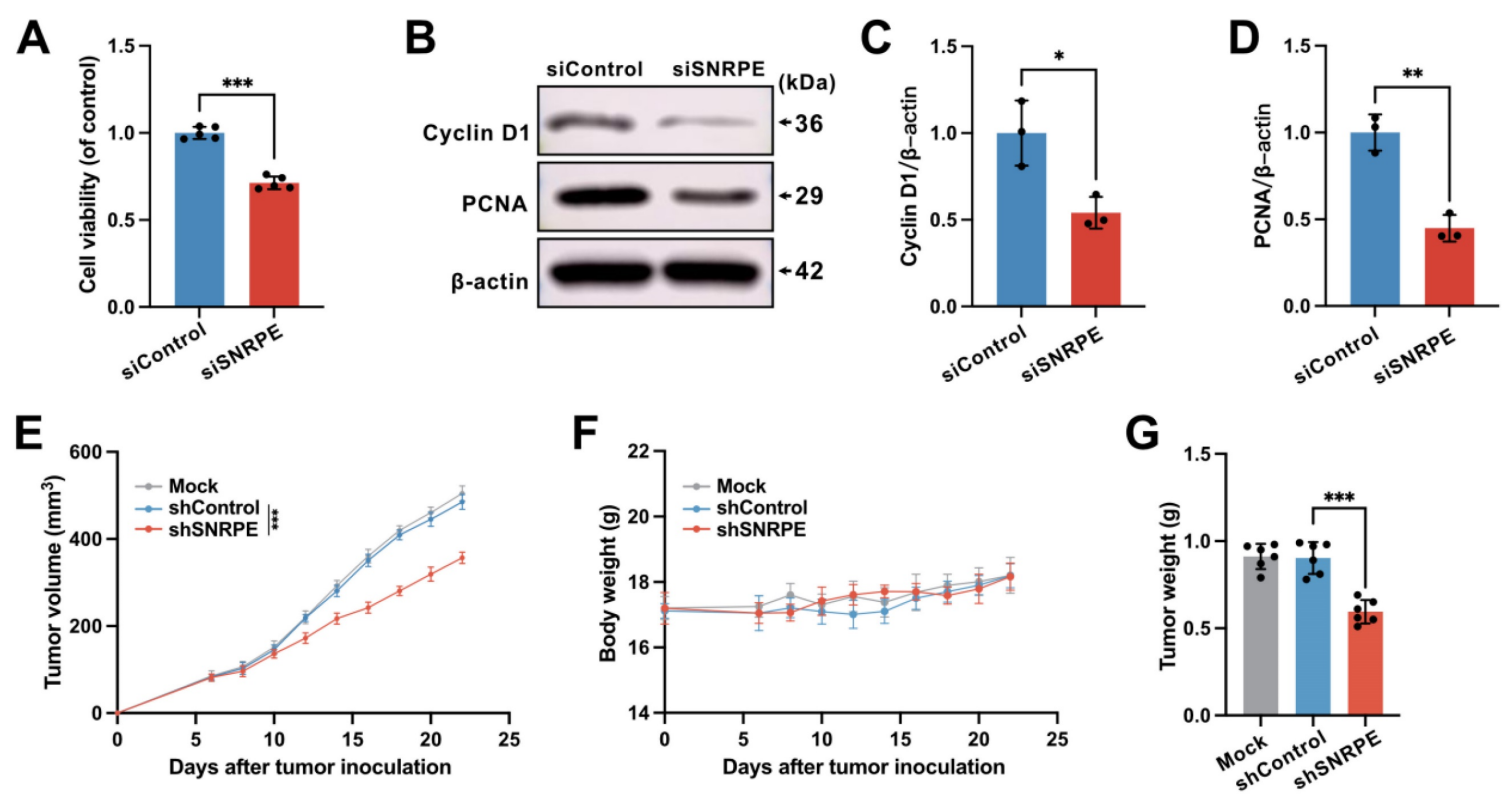
** Targeting dysregulated SNRPE in breast cancer exerts the antitumor effects. A** Cellular viability of MDA-MB-231 mammary carcinoma cells transfected with siSNRPE or siControl RNAs (n=5). **B, C, D** Probing of Cyclin D1, PCNA, and β-actin expressions through western blotting, conducted on lysates derived from mammary carcinoma cells transfected with siSNRPE or siControl RNAs. The expression levels were exhibited as relative intensity ratios normalized to β-actin (n=3). **E, F, G** Tumor volume (E), body weight (F), and tumor weight (G) of mice bearing mammary carcinoma in the Mock, shControl, and shSNRPE groups (n=6). Data are expressed as means ± SD. *P < 0.05, **P < 0.01, ***P < 0.001.

To dissect the influence of SNRPE targeting on the progression of breast cancer, we assessed the cellular viability of mammary carcinoma cells transfected with siSNRPE or siControl RNAs. Indeed, compared to the siControl RNA group, the knockdown of SNRPE significantly diminished the viability of tumor cells (Figure 2A). Subsequently, we inspected the expression levels of key proteins involved in the cell cycle process. Indeed, under the condition of siSNRPE RNA, tumor cells exhibited notably diminished level of Cyclin D1 compared to the siControl RNA group, corroborating the suppressive effect of SNRPE silencing on the cell cycle process of breast cancer cells (Figure 2BC). To further substantiate the inhibitory impact of SNRPE depleting on the progression of breast cancer, we evaluated the marker of cell proliferation. Consistently, in comparison to the siControl RNA group, the expression level of the PCNA protein in tumor cells transfected with siSNRPE RNA was markedly decreased (Figure 2BD). To evaluate the antitumor effects of targeting SNRPE *in vivo*, the mammary carcinoma xenograft murine model was established.

The mock group served as the blank control, while the shControl group served as the negative control. The results demonstrated that targeting SNRPE exhibited significant antitumor effects without affecting body weight (Figure 2EF). Consistently, compared to the shControl group, the shSNRPE group exhibited a significant reduction in tumor weight (Figure 2G). These findings confirm that SNRPE targeting can manifest antitumor effects in breast cancer.

### NK cells are activated after SNRPE targeting of breast cancer

**Figure 3:**
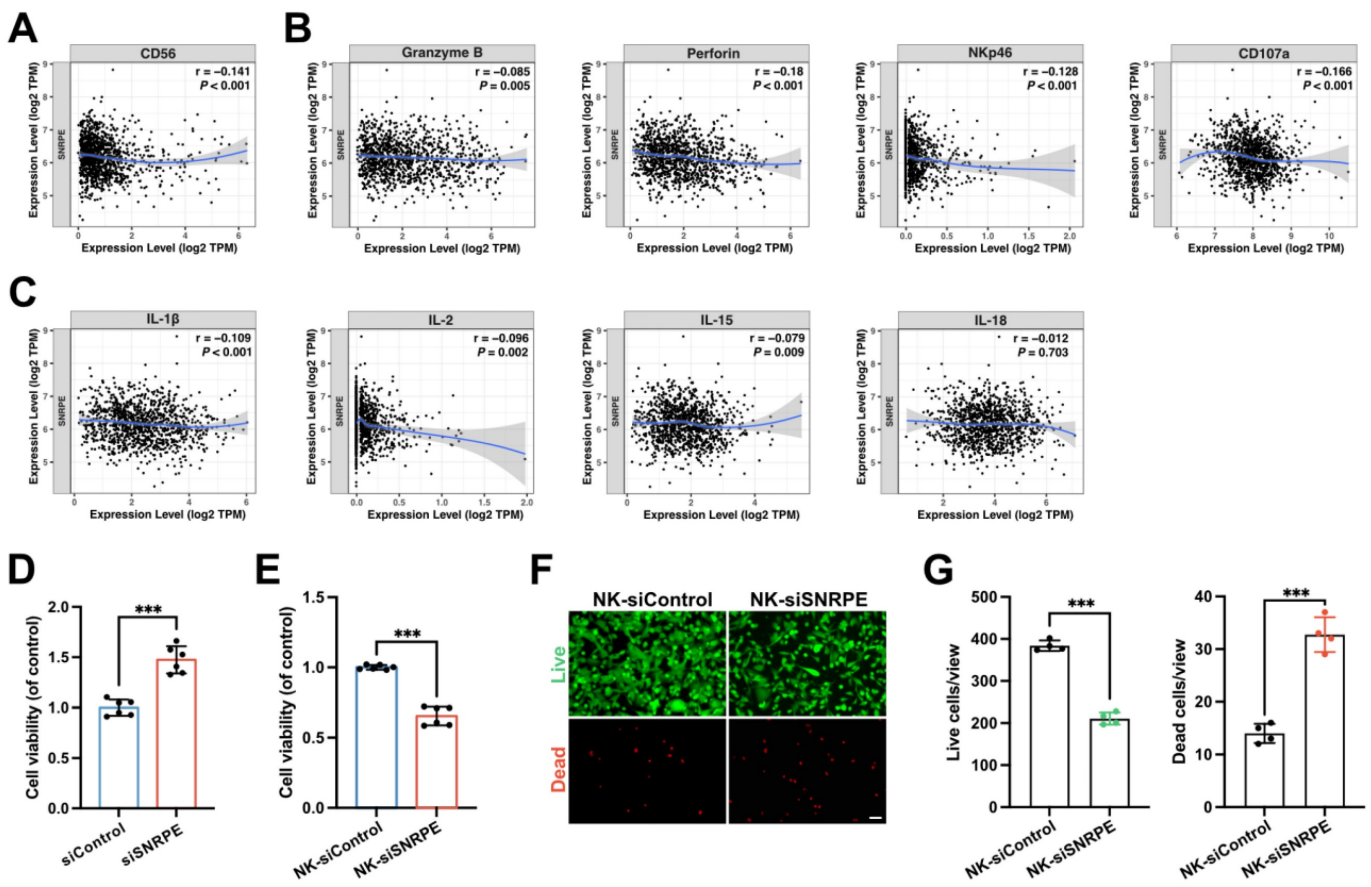
** NK cells are activated after SNRPE targeting of breast cancer. A** Analysis of the correlation between SNRPE expression and CD56 expression in BRCA (n=1100). **B** Analysis of the correlation between SNRPE expression and the expression of Granzyme B, perforin, NKp46, and CD107a in BRCA (n=1100). **C** Analysis of the correlation between SNRPE expression and the levels of the activating cytokines IL-2, IL-15, IL-18, and IL-1β in BRCA (n=1100). **D** Cellular viability of NK cells co-cultured with mammary carcinoma cells transfected with siSNRPE or siControl RNAs (n=6). **E** Mammary carcinoma cells transfected with siSNRPE or siControl RNAs were separately co-cultured with NK cells, followed by co-culturing the isolated NK cells (designated as NK-siControl or NK-siSNRPE group, respectively) with tumor cells to assess the viability of tumor cells (n=6). **F, G** Representative fluorescence images and quantification of mammary carcinoma cell samples stained for live and dead cells using Calcein-AM and propidium iodide (PI) respectively (n=4). Scale bars: 100 µm. Data are expressed as means ± SD. ***P < 0.001.

Considering the immune infiltration analysis revealed a significant negative correlation between the expression level of SNRPE and the infiltration levels of immune cells especially NK cells in breast cancer, we proceeded to investigate the impact of SNRPE targeting in tumor cells on NK cells (Figure 1K). To characterize the effect of SNRPE on the infiltration abundance of NK cells, CD56 expression was analyzed (Figure 3A). Analysis *in silico* based on TCGA breast cancer patient samples revealed that SNRPE was negatively correlated with CD56 expression in breast cancer. To investigate the effect of SNRPE on the cytotoxicity and activation status of NK cells, the expression of related markers was analyzed (Figure 3B). SNRPE was negatively correlated with the expression of Granzyme B, perforin, NKp46 and CD107a. Further, we analyzed NK-cell-related cytokines and discovered that SNRPE was negatively correlated with the levels of the activating cytokines IL-2, IL-15, IL-18, and IL-1β (Figure 3C).

In agreement with the correlation analysis *in silico*, SNRPE targeting of tumor cells amplified NK cell-mediated antitumor immunity. Mammary carcinoma cells transfected with siSNRPE or siControl RNAs were co-cultured separately with NK cells. Indeed, compared to the control group, NK cells co-cultured with siSNRPE-transfected tumor cells exhibited the significantly increased cellular viability (Figure 3D).

**Figure 4:**
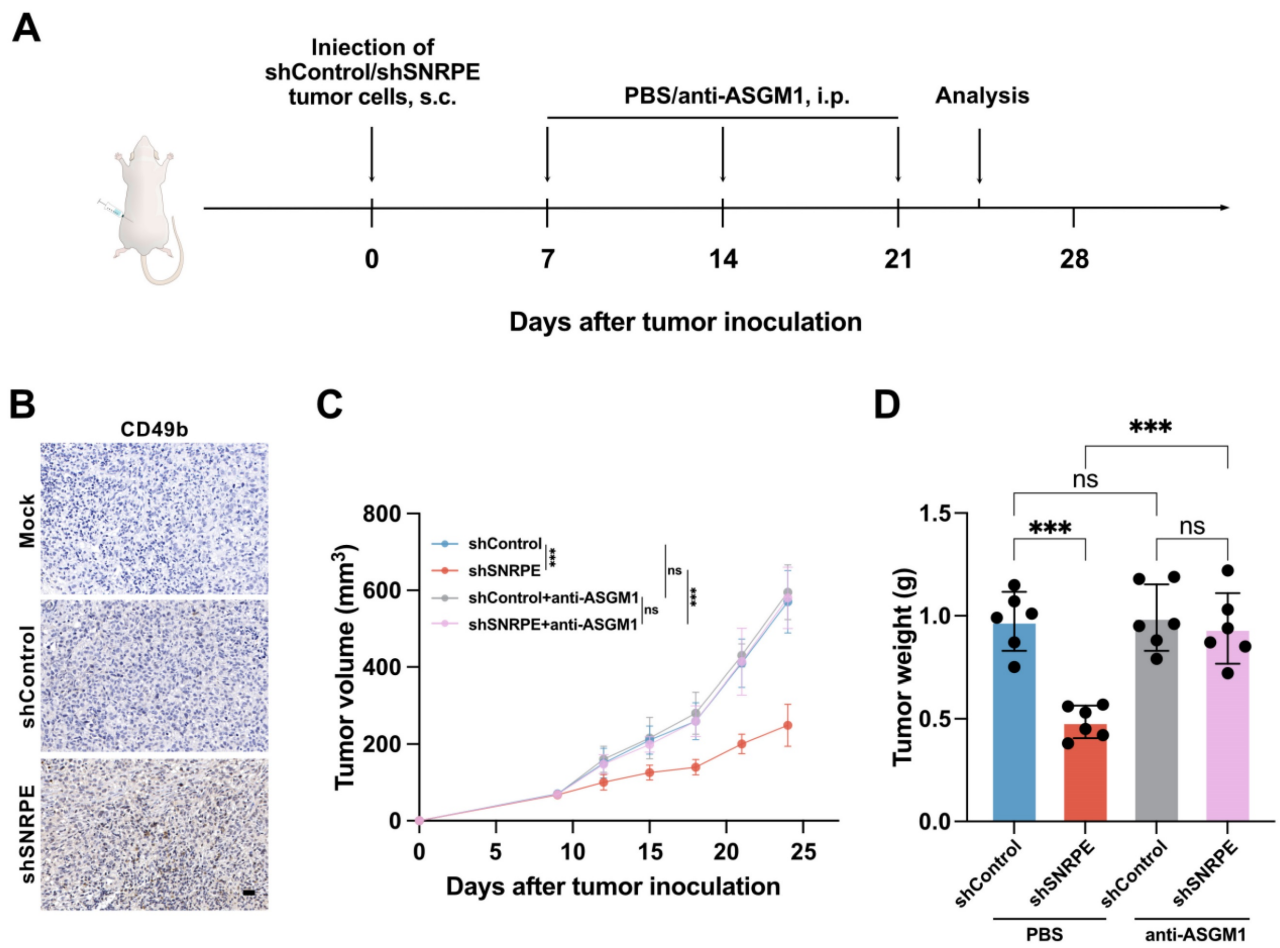
** SNRPE targeting inhibits tumor growth by activating NK cell-mediated antitumor immunity. A** Schematic illustration of experimental design for B, C and D. **B** NK cell infiltration in tumor tissues. Representative immunohistochemistry images are shown; scale bars: 20 µm. **C** Tumor volume in mice implanted with MDA-MB-231 mammary carcinoma cells. The mice were intraperitoneally injected with a nti-ASGM1 antibody for NK cell depletion (n=6). **D** Tumor weight of mice bearing mammary carcinoma (n=6). Data are expressed as means ± SD. ***P < 0.001. ns, no significant difference.

To further assess the enhanced cytotoxicity of NK cells against tumor cells, we co-cultured tumor cells transfected with siSNRPE or siControl RNAs separately with NK cells, and subsequently co-cultured the isolated NK cells with mammary carcinoma cells. Indeed, compared to the control group, mammary carcinoma cells co-cultured with enhanced NK cells exhibited the significantly decreased cellular viability (Figure 3E). Consistent results were also observed in the tumor cell samples stained with Calcein-AM and propidium iodide (PI) for live and dead cell, respectively (Figure 3F,G). Furthermore, downregulation of intratumoral SNRPE enhanced the infiltration of NK cells (Figure 4B).

### SNRPE targeting inhibits tumor growth by activating NK cell-mediated antitumor immunity

To ascertain whether SNRPE targeting suppresses the tumor growth of breast cancer via activating NK cell-mediated antitumor immunity, we utilized the NK cell depleting antibody to deplete NK cells in mice (Figure 4A). We discovered that depletion of NK cells attenuated the antitumor effects of SNRPE targeting (Figure 4CD). Hence, NK cells play a pivotal role in the enhanced antitumor immunity mediated by SNRPE targeting. These experimental findings suggest that SNRPE targeting enhances NK cell-mediated antitumor immunity.

### SNRPE targeting triggers pyroptosis of tumor cells

**Figure 5:**
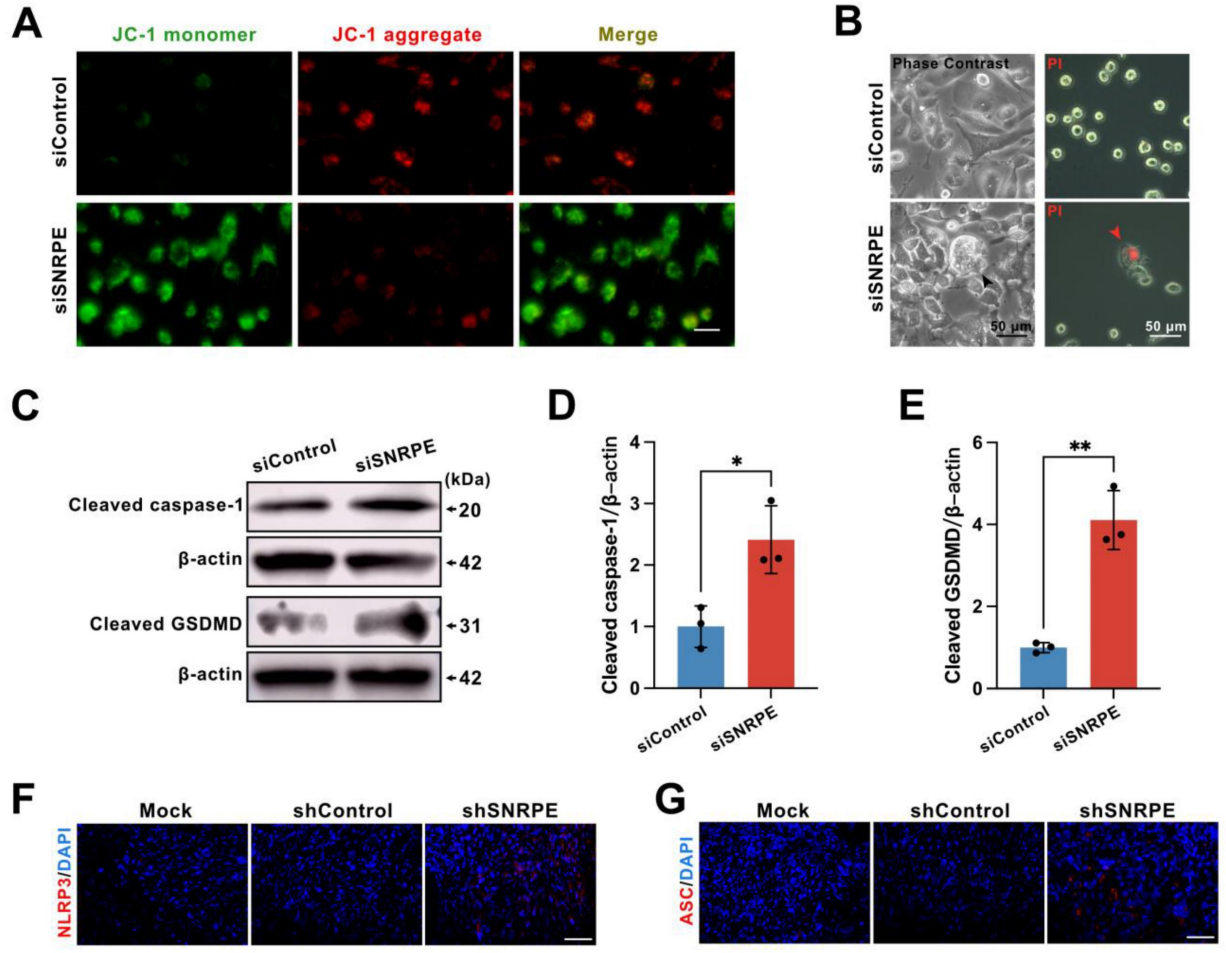
** SNRPE targeting triggers pyroptosis of tumor cells. A** Alterations in the mitochondrial membrane potential in mammary carcinoma cells. Scale bars: 200 µm. **B** Phase contrast images (left) and representative fluorescence images (right) of mammary carcinoma cells transfected with siSNRPE or siControl RNA stained with propidium iodide (PI) (red) (n=3). Scale bars: 50 µm. **C, D, E** Lysates from mammary carcinoma cells transfected with siSNRPE or siControl RNAs probed via western blots for the expression of Cleaved caspase-1, Cleaved GSDMD, and β-actin as a loading control. Relative intensity ratios normalized to β-actin (n=3). **F, G** Immunofluorescence staining of NLRP3- (red) (F) and ASC- (red) (G) in mammary carcinoma tissue sections collected from mice; scale bars: 50 µm. Data are expressed as means ± SD. *P < 0.05, **P < 0.01.

Further, the mechanism by which SNRPE targeting activates antitumor immunity was explored. Utilizing the dual fluorescent mitochondrial membrane potential assay kit (JC-1), we investigated the depolarization of the mitochondrial membrane in breast cancer cells. The red fluorescent probe in the control cells revealed a normal mitochondrial membrane potential, whereas tumor cells transfected with siSNRPE RNA exhibited green fluorescence, indicating a significant reduction in the mitochondrial potential (Figure 5A). Notably, we examined the morphological changes in tumor cells and discovered that cells transfected with siSNRPE RNA exhibited noticeable swelling and cell membrane rupture, and took up propidium iodide (PI) red, which are hallmark morphological features of pyroptosis (Figure 5B).

To ascertain whether signal transduction resulting from targeting SNRPE could induce pyroptosis, an inflammatory programmed cell death platform, we evaluated the expression levels of key proteins in the pyroptotic response. Indeed, compared to the control group, the knockdown of SNRPE fostered the expression levels of cleaved caspase-1 and cleaved GSDMD (Figure 5CDE). Immunocytochemical staining of murine mammary carcinoma tissue demonstrated that SNRPE silencing increased NLRP3 expression as well as the formation of the characteristic ASC specks (Figure 5FG). Collectively, these data suggest that SNRPE targeting triggers pyroptosis in tumor cells.

### Antitumor immunity activated by SNRPE targeting is pyroptosis-dependent

**Figure 6:**
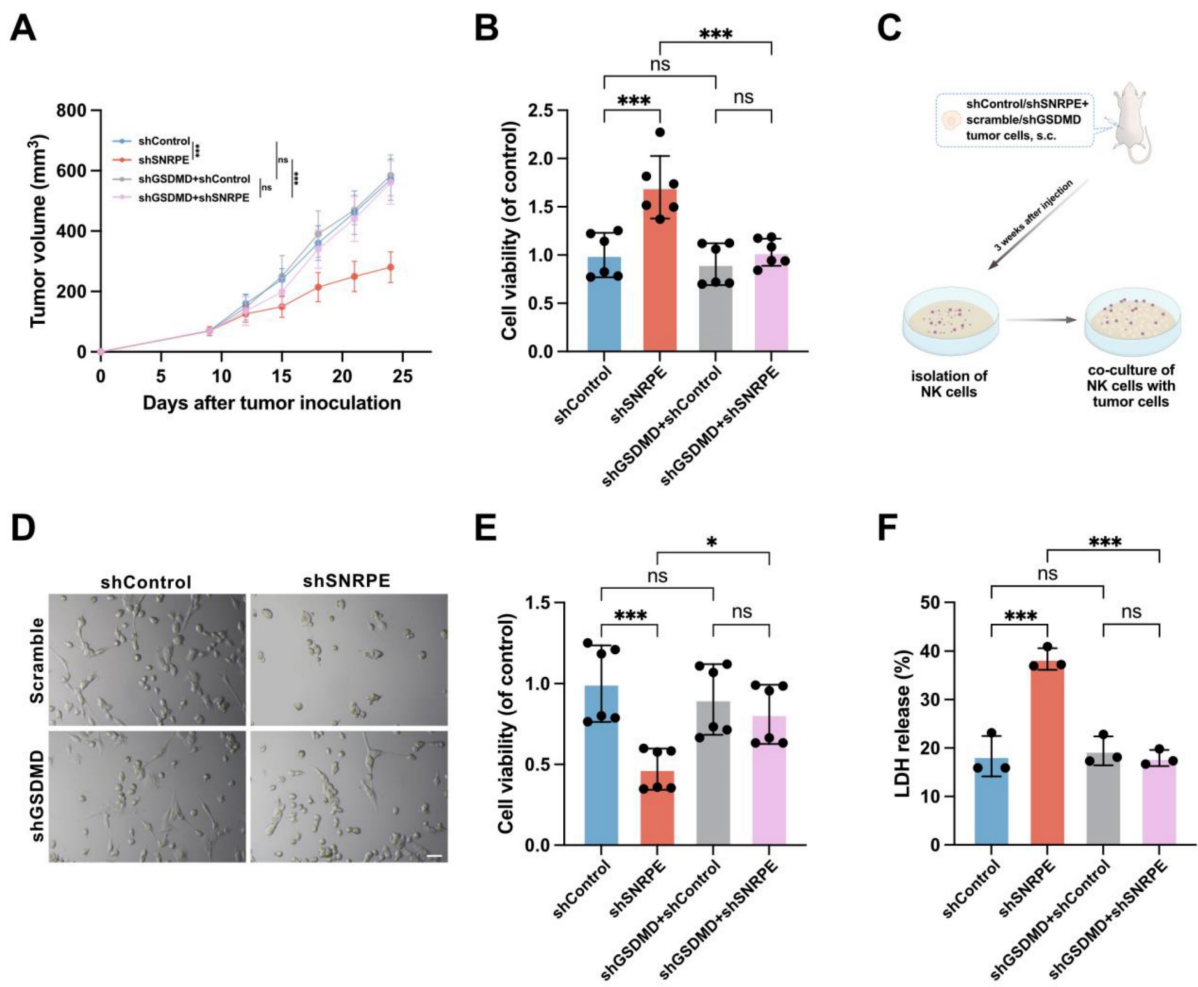
** Antitumor immunity activated by SNRPE targeting is pyroptosis-dependent. A** Tumor volume in mice implanted with shControl/shSNRPE+scrambled/shGSDMD mammary carcinoma cells (n=6). **B** Cell viability of NK cells isolated from mammary tumor-bearing mice (n=6). **C** Experimental design. Three weeks after tumor inoculation, NK cells isolated from mice bearing mammary carcinoma were co-cultured with MDA-MB-231 mammary carcinoma cells. **D** Representative images showing death induced in MDA-MB-231 cells by NK cells isolated from mice bearing mammary carcinoma (n=6). Scale bars: 100 µm. **E** Assessment of the viability of MDA-MB-231 cells co-cultured with isolated NK cells using the CCK8 assay (n=6). **F** Evaluation of the cytotoxic effect of isolated NK cells against mammary carcinoma cells using the LDH release assay (n=3). Data are expressed as means ± SD. *P < 0.05, ***P < 0.001. ns, no significant difference.

To ascertain whether GSDMD plays a major role in SNRPE targeting-induced pyroptosis for amplifying antitumor immunity, we employed short hairpin RNAs (shGSDMD RNA) to knock down the expression of GSDMD, with scrambled shRNA used as a control. Given that MDA-MB-231 cells express high levels of GSDMD (Figure S1B), we established a murine model using scramble or shGSDMD MDA-MB-231 cells for further investigation. SNRPE targeting had minimal effects in mice implanted with the shGSDMD cell line compared to mice implanted with a cell line expressing a control scrambled shRNA (Figure 6AB). This signifies that the knockdown of GSDMD reversed the tumor-suppressive and immune-enhancing effects of SNRPE targeting.

Moreover, co-culture experiments involving isolated NK cells and tumor cells revealed that SNRPE targeting heightened the cytotoxicity of NK cells, while GSDMD knockdown disrupted the SNRPE silencing-induced augmentation of antitumor immunity and inhibition of tumor growth (Figure 6CDEF). These data underscore that SNRPE targeting enhances antitumor immunity in breast cancer by inducing GSDMD-mediated pyroptosis of tumor cells.

### ROS are required for the induction of pyroptosis by SNRPE targeting

**Figure 7:**
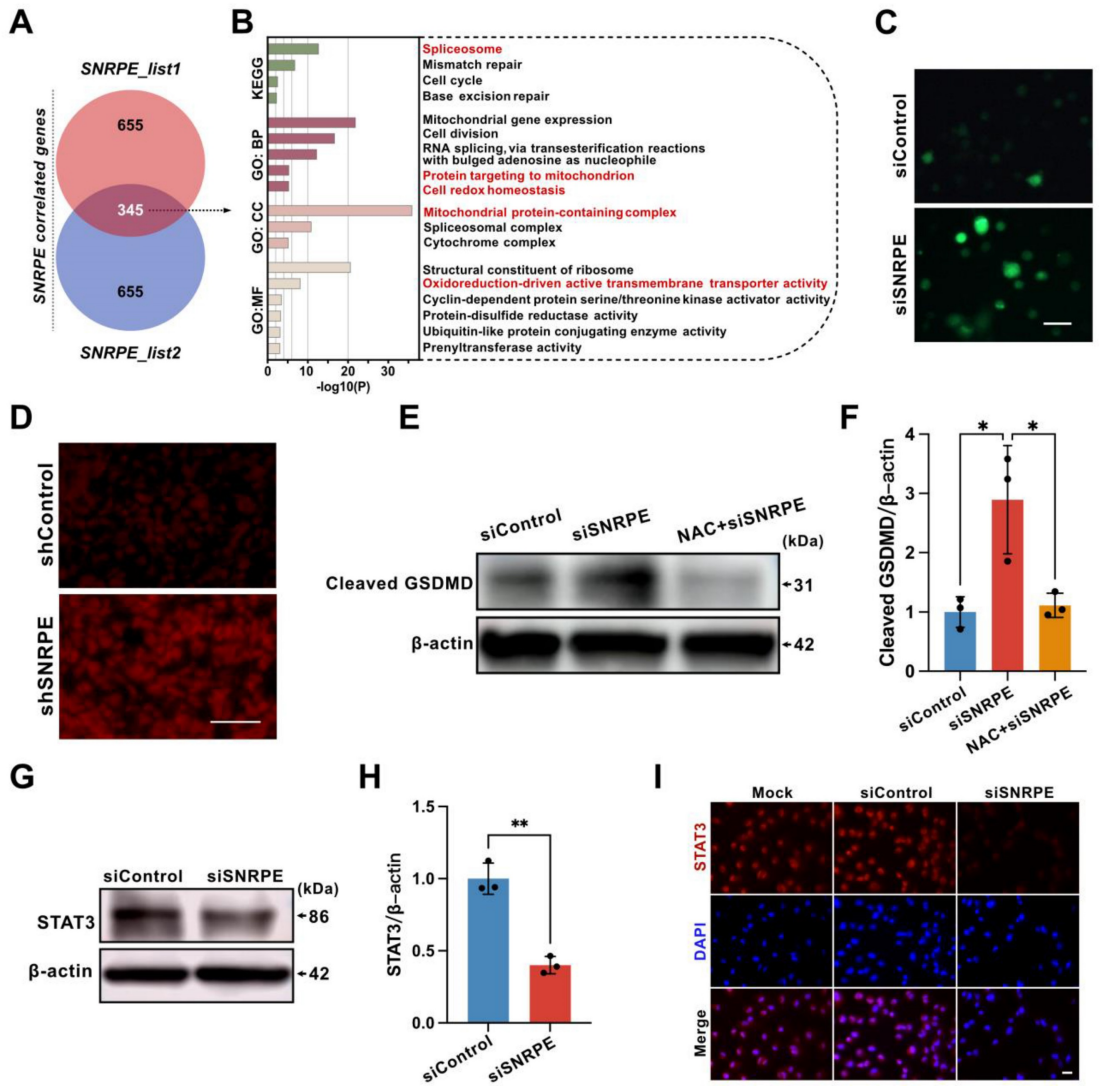
** ROS are required for the induction of pyroptosis by SNRPE targeting. A** Identification of 345 genes correlated with SNRPE via Venn diagram analysis. **B** GO and KEGG enrichment analysis based on SNRPE expression in breast cancer. **C** Representative fluorescence images of ROS in MDA-MB-231 mammary carcinoma cells transfected with siSNRPE or siControl RNA, traced using DCFH-DA fluorescent probe (n=6). Scale bars: 50 µm. **D** Representative fluorescence images of dihydroethidium (DHE) staining of breast cancer tumor tissue. (n=6). Scale bars: 100 µm. **E, F** Western blotting analysis of lysates from MDA-MB-231 mammary carcinoma cells untreated or primed with NAC, transfected with siSNRPE or siControl RNAs. Quantification of Cleaved-GSDMD normalized to β-actin levels (n = 3). **G, H** Western blotting of lysates procured from breast cancer cells transfected with siSNRPE or siControl RNAs and quantification of STAT3 relative to β-actin levels (n=3). **I** Representative immunofluorescence images of STAT3 (red) and DAPI (blue) in mammary carcinoma cells transfected with siSNRPE or siControl RNAs (n=4). Scale bars: 25 µm. Data are expressed as means ± SD. *P < 0.05, **P < 0.01.

To further elucidate how SNRPE targeting triggers pyrolysis of mammary carcinoma cells, the pathway analyses were conducted* in silico*. We performed gene selection using cBioPortal and GEPIA2 to identify significantly correlated genes with SNRPE expression, resulting in two gene lists, list1 and list2 (Figure S2). Using a Venn diagram, we identified 345 common genes from these two lists (Figure 7A), and subsequently conducted KEGG and GO enrichment analyses (Figure 7B and Figure S3). The KEGG analysis revealed that pathways such as "spliceosome", "mismatch repair", "cell cycle", and "base excision repair" may be involved in the impact of SNRPE on breast cancer pathogenesis. The GO enrichment analysis primarily highlighted biological processes related to "mitochondrial gene expression", "cell division", "RNA splicing", "protein targeting to mitochondrion", "cell redox homeostasis", "mitochondrial protein-containing complex", "cytochrome complex", "structural constituent of ribosome", "oxidoreduction-driven active transmembrane transporter activity", "cyclin-dependent protein serine/threonine kinase activator activity", "protein-disulfide reductase activity", "ubiquitin-like protein conjugating enzyme activity", and "prenyltransferase activity".

In light of the terms enriched in the pathway analysis, we proceeded to transfect MDA-MB-231 mammary carcinoma cells with siSNRPE or siControl RNAs, subsequently evaluating the ROS levels using DCFH-DA (Figure 7C). In fact, compared with the siControl RNA group, tumor cells under the siSNRPE RNA condition manifested a marked increase in ROS levels. Additionally, we proceeded to validate this phenomenon *in vivo*. Similarly, SNRPE targeting led to an augmentation of ROS levels at the mouse tumor site (Figure 7D).

Having confirmed that SNRPE targeting boosted ROS generation and triggered pyroptosis, we then conditioned tumor cells treated with NAC (a ROS inhibitor) using siSNRPE or siControl RNAs to determine the necessity of ROS in the pyroptotic response induced by SNRPE targeting (Figure 7EF). Indeed, tumor cells transfected with siSNRPE exhibited elevated level of cleaved-GSDMD. Notably, SNRPE targeting was unable to enhance the expression level of cleaved-GSDMD in tumor cells treated with NAC. These data underscore that the induction of the pyroptotic response in mammary carcinoma cells by targeting SNRPE is ROS-dependent.

Signal transducer and activator of transcription 3 (STAT3) is intricately associated with the regulation of the electron transport chain in mitochondria and the modulation of mitochondrial ROS^28^. In addition, STAT3 can augment the activity of mitochondrial electron transport chain complex I to curtail ROS production, irrespective of its conventional function as a nuclear transcription factor^29^. Based on this, we postulated that SNRPE targeting might impact STAT3, consequently leading to elevated levels of ROS. Indeed, compared to the siControl RNA group, the level of STAT3 in tumor cells transfected with siSNRPE RNA was markedly decreased (Figure 7GH). Consistently, the immunofluorescent staining of breast cancer cells with SNRPE knockdown revealed significantly decreased level of STAT3 (Figure 7I). These findings show that the depletion of SNRPE may boost ROS levels in mammary carcinoma cells by targeting STAT3.

## Discussion

In this study, we revealed a negative correlation between the core spliceosomal component SNRPE and antitumor immunity in breast cancer. Our data demonstrated SNRPE targeting amplified NK cell-mediated antitumor immunity in the tumor microenvironment by triggering pyroptosis of tumor cells *in vivo*, and modulated pyroptosis of tumor cells in a ROS-dependent manner, providing important mechanistic insights into spliceosome targeting therapy for enhanced antitumor immunity in inflammatory tumors.

The spliceosome participates in the removal of introns from pre-mRNA and generates multiple mature mRNA isoforms^9^, ^10^. SNRPE, as the core spliceosomal component, is associated with tumor progression^11^. Targeting of splicing suppresses some cancers, but how it kills tumors remains unclear^11^, ^30^-^32^. Early studies emphasized the significance of aberrant protein products arising from incorrect splicing. Recent research has shifted its focus towards the accumulation of endogenous mis-spliced RNAs triggered by spliceosome inhibition and the activation of antiviral pathways within tumor cells^14^. In contrast, here, we discover the association of SNRPE with the immune-suppressed microenvironment in breast cancer. Our findings suggest that targeting splicing not only leads to tumor cell death but also activates immune cells, thereby enhancing antitumor immunity, revealing insights into spliceosome-immune crosstalk.

Persistent inflammation plays a crucial role in the disease progression of breast cancer, both locally and systemically. Modulating the tumor's immune microenvironment can have an impact on the development, metastasis, and treatment of breast cancer^15^. Emerging evidence has indicated that immune reactivation is a prerequisite for achieving successful tumor eradication^16^, ^18^, ^33^. Here, we discover that SNRPE targeting directly modulates the tumor microenvironment. SNRPE targeting of tumor cells activates NK cell-mediated antitumor immunity. It may improve the limited efficacy of T cell-based anti-PD-1 therapy in enhancing anti-tumor immunity^34^. Furthermore, we unveil novel roles and mechanisms of SNRPE targeting in tumors. Previous studies primarily focused on the impact of splicing inhibition on cancer cell proliferation, migration, and the cell cycle^11^, ^13^. Here, we discover that SNRPE targeting induces pyroptotic cell death in breast cancer, which was distinct from apoptosis, subsequently activating antitumor immune responses. This fresh perspective and mechanism imply that spliceosome targeting may influence distinct phenotypes in different tumor tissues, providing new evidence for the interaction between the spliceosome-targeted tumors and the host immune system. Differing from studies focusing solely on suppressing tumor cells or boosting immune cells^35^-^37^, the current research indicates that spliceosome-targeted therapy not only directly inhibits tumors but also enhances antitumor immunity, thereby exerting remarkable antitumor effects.

The question arises as to how the SNRPE targeting induces pyroptosis through the cleavage of caspase-1/GSDMD in tumor cells. Emerging evidence has indicated that reactive oxygen species (ROS) serve as the primary mediators of pyroptosis^19^, ^38^, ^39^. We speculated that SNRPE targeting might induce pyroptosis in tumor cells by boosting ROS levels. However, the putative regulation of ROS by SNRPE remains unclear. We discovered that SNRPE targeting enhanced ROS generation and induced caspase-1/GSDMD-mediated pyroptosis in mammary carcinoma cells. Notably, the antioxidant N-acetyl-l-cysteine (NAC), an ROS inhibitor, blocked pyroptosis induced by SNRPE silencing. This rescue phenomenon demonstrated that SNRPE targeting triggered the pyroptosis in a ROS-dependent manner. The relationship between STAT3 and ROS is controversial, as some studies suggest that STAT3 acts as an upstream mediator of ROS^40^, while others indicate that ROS activates STAT3^41^. Mitochondrial STAT3 restricted the production of mitochondrial ROS in response to stress injury^40^. Conversely, knocking down STAT3 increased ROS production in breast cancer cells^42^. In a study on cardiac ischemia-reperfusion, STAT3 enhances the activity of mitochondrial electron transport chain complex I to decrease production of ROS, independent of its canonical activity as a nuclear transcription factor^29^. In our study, the depletion of SNRPE might induce ROS generation by inhibiting STAT3, as the knockdown of SNRPE decreased STAT3 level and increased production of ROS in breast cancer cells. Building upon these findings, we reasoned that the depletion of SNRPE might trigger ROS-mediated pyroptosis through targeting STAT3 in tumor cells. Further comprehensive investigations are warranted to elucidate the regulatory mechanism by which SNRPE modulates STAT3. In light of SNRPE is a core spliceosomal component, a reasonable and plausible assumption can be made that the dysregulation of SNRPE disrupts the proper splicing of pre-mRNA, resulting in the aberrant formation of STAT3 mRNA.

Although our data support the hypothesis that the potential mechanisms of SNRPE targeting to trigger pyroptotic cell death are related to ROS generation, the necessity of targeting STAT3 to increase ROS production in pyroptosis mediated by depletion of SNRPE warrants further investigation. In addition, we have demonstrated that NK cells make a pivotal contribution to the enhanced antitumor immunity by SNRPE targeting of mammary carcinoma cells through triggering pyroptosis. However, it remains to be explored whether other types of immune cells play significant roles in the pathway from pyroptosis of tumor cells to activation of NK cells. Taken together, this study offers new insights into the interaction between spliceosome-targeted tumors and host immunity, highlighting the spliceosome targeting to trigger pyroptosis as a comprehensive therapeutic strategy for enhanced antitumor immunity in breast cancer.

## Supplementary Material

Supplementary figures and tables.

## Abbreviations

DMFS: distant metastasis-free Survival
DSS: disease-specific survival
GEPIA: gene expression profiling interactive analysis
GSDMD: gasdermin D
HRP: horseradish peroxidase
ns: no significant difference
OS: overall survival
PCNA: proliferating cell nuclear antigen
PFS: progression-free survival
pre-mRNA: precursor mRNA
RFS: recurrence-free survival
RNAi: RNA interference
RNA-seq: RNA sequencing
siControl: transfected with scrambled siRNAs
siRNA: small-interfering RNA
siSNRPE: transfected with siRNAs to suppress the SNRPE gene
SNRPE: small nuclear ribonucleoprotein polypeptide E
TCGA: the cancer genome atlas
