# Supplementary material for: Targeting SNRPE to Induce Pyroptosis Enhances Antitumor Immunity in Breast Cancer
Source: Int J Med Sci. 2025 Apr 28;22(10):2419–33. doi: 10.7150/ijms.109171 (PMC12080576; doi:10.7150/ijms.109171)
Supplement: Supplementary file 1 — Supplementary figures and tables. [file ijmsv22p2419s1.pdf]

Supplementary Material

Targeting SNRPE to induce pyroptosis enhances antitumor immunity in breast cancer

Supplementary Figure 1. The respective protein expression levels of SNRPE in MCF7 and MCF10 A cells; Transcript levels of GSDMD in MDA-MB-231 cells.

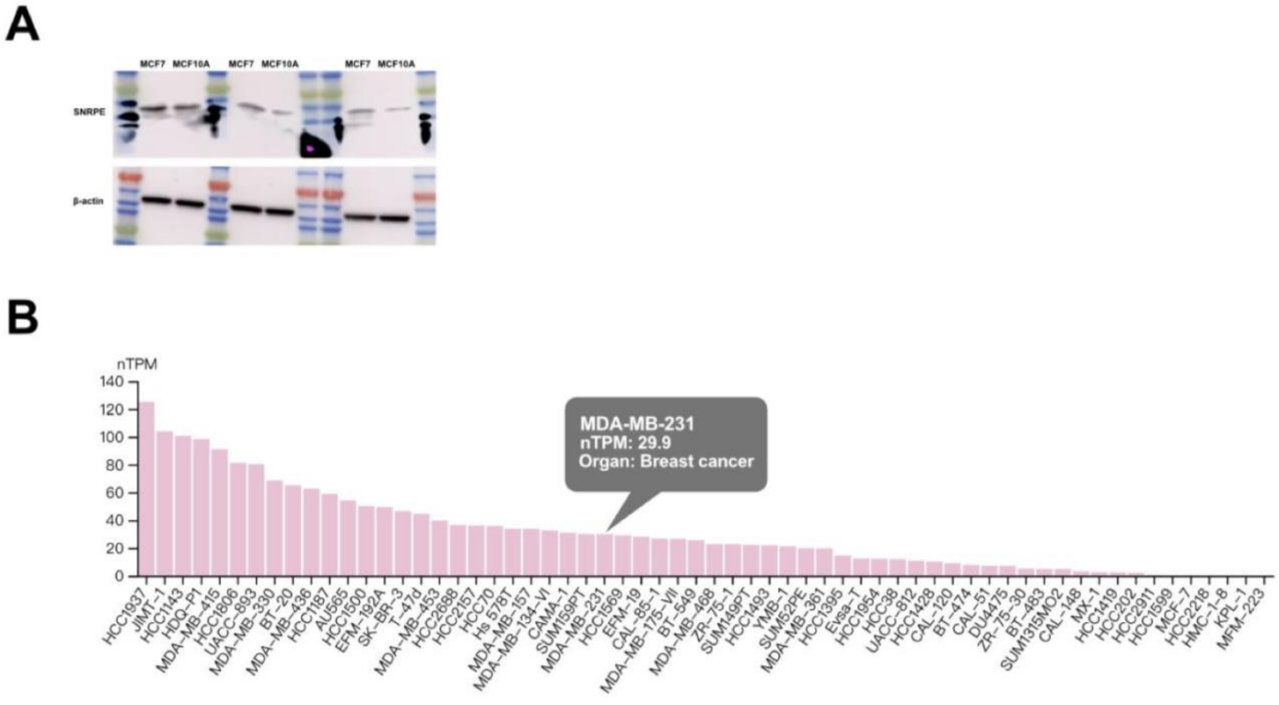

Supplementary Figure 2. The lists of genes used for enrichment analysis in breast cancer.

A

List 1 (cBioPortal)

	Gene		Gene		Gene		Gene		Gene		Gene		Gene
1	**TMEM183A**	51	**ARL8A**	101	**TOMM5**	151	**CCDC167**	201	**PALM2AKAP2**	251	**PTRH2**	301	**RC3H2**
2	**COA6**	52	**GNPAT**	102	**MGST3**	152	**MYO9A**	202	**RFC2**	252	**TIMM9**	302	**SAMD8**
3	**RBM34**	53	**AKAP13**	103	**MORN2**	153	**PCNX1**	203	**GTF3C6**	253	**GNA11**	303	**BUD31**
4	**H3-3A**	54	**LSM 5.00**	104	**VPS13C**	154	**TBC1D2B**	204	**NOP16**	254	**MRPL1**	304	**SNRPA**
5	**CACYBP**	55	**MAGOHB**	105	**ZNF366**	155	**C1ORF43**	205	**PSMG2**	255	**NACA4P**	305	**FAM168A**
6	**MIX23**	56	**SNRPD1**	106	**MYOF**	156	**PPIH**	206	**RPL24**	256	**SF3B6**	306	**ATP5PF**
7	**TIMM17A**	57	**HERC2**	107	**TXNDC17**	157	**GAS5**	207	**METTL1**	257	**H2AZ1**	307	**PLBD2**
8	**GLRX2**	58	**HSPE1**	108	**GGCT**	158	**ZBTB40**	208	**TRIM56**	258	**TBC1D7**	308	**BPNT1**
9	**CNIH4**	59	**TMEM258**	109	**HNRNPC**	159	**VPS13D**	209	**SARNP**	259	**HIPK1**	309	**KMT2D**
10	**TMEM81**	60	**SEM1**	110	**SRSF3**	160	**CHCHD1**	210	**DENND4A**	260	**NDUFA12**	310	**RNPEP**
11	**MRPL9**	61	**POLR2H**	111	**CHTOP**	161	**CBX3**	211	**EBPL**	261	**RPS21**	311	**PKD2**
12	**CCT3**	62	**ILF2**	112	**GARIN2**	162	**ZNF672**	212	**CACNA2D1**	262	**APH1A**	312	**AKAP10**
13	**PRDX6**	63	**RBIS**	113	**ELK3**	163	**HNRNPA1**	213	**POLR2J**	263	**PSMA3**	313	**NDUFB6**
14	**PFDN2**	64	**LSM 2.00**	114	**PDCD5**	164	**ZZEF1**	214	**CKS2**	264	**S100A11**	314	**PDCL3**
15	**ACBD6**	65	**ATP5MJ**	115	**NENF**	165	**SNAPIN**	215	**SFXN4**	265	**MRPL18**	315	**SNORD116-4**
16	**PSMD4**	66	**MRPL47**	116	**METTL5**	166	**BANF1**	216	**ATP5F1C**	266	**LSM 8.00**	316	**PRDM2**
17	**PSMB4**	67	**UCK2**	117	**TFB2M**	167	**TNS1**	217	**POMP**	267	**NR2C2AP**	317	**TMCO1**
18	**DAP3**	68	**LEPROT**	118	**RAPGEF1**	168	**PARD3B**	218	**TBCA**	268	**UBQLN4**	318	**ATP5MC1**
19	**COP 1**	69	**RAB25**	119	**BLTP1**	169	**DYNLT1**	219	**SRSF7**	269	**MARCHF8**	319	**CTDSP2**
20	**FH**	70	**NDUFS2**	120	**NOP58**	170	**NDUFB11**	220	**DYNLL1**	270	**HNRNPAB**	320	**RGPD4**
21	**UFC1**	71	**CBX6**	121	**SLIRP**	171	**ENY2**	221	**SLC30A4**	271	**ZNF695**	321	**EXOSC1**
22	**TBCE**	72	**TPRKB**	122	**C18ORF21**	172	**COX7C**	222	**NDUFS5**	272	**LBHD1**	322	**TSACC**
23	**UBE2T**	73	**LAMTOR2**	123	**TOR3A**	173	**TRIAP1**	223	**ASXL2**	273	**MASP2**	323	**SECISBP2L**
24	**FLAD1**	74	**TSEN15**	124	**B4GALT3**	174	**NIT2**	224	**MRPS24**	274	**JPT1**	324	**DNAH14**
25	**HAX1**	75	**NAXE**	125	**TTBK2**	175	**RPP21**	225	**ARV1**	275	**BOLA2**	325	**NUF2**
26	**SNRPF**	76	**CHD2**	126	**NME1**	176	**WDFY3-AS2**	226	**SNX29**	276	**MRPS33**	326	**KIF13A**
27	**SCNM1**	77	**MRPL55**	127	**TARDBPP3**	177	**HIPK3**	227	**ERCC6L2**	277	**MSTO1**	327	**TIMM10**
28	**LYPLAL1**	78	**DEDD**	128	**GEMIN6**	178	**USP21**	228	**ZNF699**	278	**GCC2**	328	**NCOA1**
29	**RABIF**	79	**C1ORF35**	129	**ATP5MF**	179	**KIRREL1**	229	**RPL30**	279	**TRMT112**	329	**BOD1L1**
30	**KRTCAP2**	80	**PMF1**	130	**RNF187**	180	**TMEM14B**	230	**SETX**	280	**PFDN6**	330	**CSNK2B**
31	**CKS1B**	81	**UBE2Q1**	131	**SMIM26**	181	**SLC50A1**	231	**UBL5**	281	**EVI5**	331	**PREX2**
32	**SSBP1**	82	**NFATC2**	132	**UXT**	182	**USF1**	232	**NDUFAB1**	282	**PIKFYVE**	332	**NME2**
33	**C1ORF131**	83	**AHNAK**	133	**PSMA2**	183	**MYCBP2**	233	**NVL**	283	**ELOC**	333	**CENPW**
34	**SNRPG**	84	**SCAMP3**	134	**TIPRL**	184	**AGO4**	234	**LAGE3**	284	**NDUFB3**	334	**RMI2**
35	**MRPL24**	85	**SNRPGP15**	135	**TRMT10C**	185	**COX7A2**	235	**SUMO1**	285	**PPP1R14B**	335	**YAE1**
36	**SF3B4**	86	**HDGF**	136	**SNRPD2**	186	**EIF2D**	236	**GPRIN3**	286	**PLEKHM3**	336	**RGP1**
37	**MTX1**	87	**SDHC**	137	**ARPC5**	187	**NEK2**	237	**SNRPB2**	287	**EMG1**	337	**RPP38**
38	**VPS72**	88	**NOPCHAP1**	138	**ZBTB38**	188	**MRPS21**	238	**RORA**	288	**RRP15**	338	**ZNF692**
39	**SSR2**	89	**GGPS1**	139	**UBR4**	189	**DUSP12**	239	**METTL18**	289	**HIVEP3**	339	**JAK1**

40	**SRP9**	90	**MRPS14**	140	**JMJD4**	190	**TAGLN2**	240	**SLC12A6**	290	**ATP5PO**	340	**VPS39**
41	**MRPS17**	91	**NTPCR**	141	**THOC7**	191	**SYNE1**	241	**WDFY3**	291	**RAB4A**	341	**UMPS**
42	**JTB**	92	**LSM 3.00**	142	**CLCN6**	192	**BRIX1**	242	**MAST4**	292	**GTF2H5**	342	**IQSEC1**
43	**TMEM9**	93	**ENSA**	143	**CREBRF**	193	**MRPL22**	243	**RTRAF**	293	**PDSS1**	343	**UQCC2**
44	**ATP5MK**	94	**AIDA**	144	**EFCAB14**	194	**COX7B**	244	**COX6B1**	294	**RGPD3**	344	**CENPH**
45	**DPY30**	95	**TOMM6**	145	**BOLA1**	195	**LUZP1**	245	**BMP2K**	295	**SS18L2**	345	**PRRC2B**
46	**PRCC**	96	**SEC61G**	146	**MINDY2**	196	**TOMM20**	246	**RPF2**	296	**DUSP23**	346	**TGFBRAP1**
47	**HERC1**	97	**HERC3**	147	**RAPGEF2**	197	**PTTG1**	247	**CHD9**	297	**RPL35A**	347	**UQCRB**
48	**ARF1**	98	**MRPL51**	148	**MYO5A**	198	**STX6**	248	**TRIM11**	298	**NUCKS1**	348	**MRPL17**
49	**SNRPC**	99	**ERH**	149	**FDPS**	199	**PPIA**	249	**MGAT5**	299	**SLIT2**	349	**FICD**
50	**PIGC**	100	**ZNRF2P1**	150	**RPL26L1**	200	**TXN**	250	**SPTBN1**	300	**DDI2**	350	**TXNL4A**

List 1 (cBioPortal)

	Gene		Gene		Gene		Gene		Gene		Gene		Gene
351	**NHP2**	401	**PAM16**	451	**SH2B3**	501	**RUNX1T1**	551	**MAGI1**	601	**PYCR2**	651	**RALGAPA2**
352	**MIGA1**	402	**ABRACL**	452	**RAB5IF**	502	**UBA52**	552	**DGUOK**	602	**NUDT5**	652	**TMEM245**
353	**RNASEH2A**	403	**PAXIP1-AS2**	453	**LMTK2**	503	**TMSB10**	553	**PLEKHM1**	603	**ATP5PD**	653	**RPL37**
354	**EFNA4**	404	**HECTD4**	454	**PSMC1**	504	**FBL**	554	**NHSL2**	604	**PACC1**	654	**ATG2B**
355	**NDUFA1**	405	**N4BP2**	455	**ZBTB4**	505	**POP7**	555	**NDUFA4**	605	**PSMA4**	655	**IPO9**
356	**RFC4**	406	**MRPL36**	456	**RPP30**	506	**FUT11**	556	**PTTG3P**	606	**SPATA13**	656	**GAPDH**
357	**PCDHGB7**	407	**TMEM147**	457	**CDKN3**	507	**CEP104**	557	**TADA1**	607	**ALG3**	657	**PCDHGB6**
358	**SPEN**	408	**BOLA3**	458	**MRPS9**	508	**HEBP2**	558	**PLPP3**	608	**EIF5AL1**	658	**PCLAF**
359	**PSMB1**	409	**CBX7**	459	**BMPR2**	509	**GTF2A2**	559	**LRR1**	609	**PRELID1**	659	**PRKG1**
360	**RNF111**	410	**RPL38**	460	**NABP2**	510	**SLC16A7**	560	**GAR1**	610	**SLC35E2A**	660	**AP1S1**
361	**EXOC6B**	411	**RPS24**	461	**UQCRH**	511	**EXOSC5**	561	**TIMM17B**	611	**NDUFB9**	661	**TATDN3**
362	**MTMR3**	412	**CEP85L**	462	**NACC2**	512	**PXK**	562	**KLF13**	612	**EIF6**	662	**HEG1**
363	**ATP5F1E**	413	**IMMP1L**	463	**DMXL1**	513	**PPTC7**	563	**PSMB7**	613	**MEMO1**	663	**NDUFV2**
364	**RPL39**	414	**C1ORF112**	464	**NDUFB1**	514	**RPL22L1**	564	**MRPS16**	614	**MAP3K2**	664	**NDUFA8**
365	**HNRNPU**	415	**FAM114A1**	465	**BICC1**	515	**NATD1**	565	**PIPSL**	615	**WDR44**	665	**MRPL58**
366	**PA2G4**	416	**MRPL48**	466	**MRPL13**	516	**SNORD116-20**	566	**PTPRG**	616	**CASS4**	666	**LIN9**
367	**SPHAR**	417	**MPC2**	467	**POLR1C**	517	**SMCR8**	567	**RBFOX2**	617	**UQCRQ**	667	**NDUFB4**
368	**ATXN1L**	418	**CCDC59**	468	**DDR2**	518	**APOO**	568	**SH3BP5L**	618	**KLHL11**	668	**WDR74**
369	**TCP11L2**	419	**ZNF106**	469	**SF3B5**	519	**PUS1**	569	**NOP56**	619	**DTL**	669	**MRPL15**
370	**TGOLN2**	420	**ZEB2**	470	**MRPL3**	520	**NDUFS3**	570	**PWAR5**	620	**NDUFA9**	670	**MSMP**
371	**PIN4**	421	**MTMR10**	471	**NDST2**	521	**MTRES1**	571	**MEF2A**	621	**LRP1**	671	**DNAJB14**
372	**PLXNC1**	422	**ALKBH2**	472	**LSM 4.00**	522	**GPATCH8**	572	**GTF2A1**	622	**COX6A1**	672	**SELENOH**
373	**PFDN4**	423	**EXOSC8**	473	**MTFR2**	523	**MIS18A**	573	**CCDC186**	623	**SEC61B**	673	**CARF**
374	**MAPKBP1**	424	**NXT1**	474	**DNAAF9**	524	**C1ORF74**	574	**CLK2**	624	**RPS2**	674	**MBD5**
375	**HDDC2**	425	**FAT4**	475	**ANAPC7**	525	**COX16**	575	**RAPGEF6**	625	**UBE2C**	675	**TECPR1**
376	**TSHZ2**	426	**HINT1**	476	**EBP**	526	**MAP3K3**	576	**PSENEN**	626	**EP300**	676	**GCNT4**
377	**DPP8**	427	**P2RX7**	477	**ITGB3**	527	**CENPL**	577	**LILRA1**	627	**COX5A**	677	**BAZ2A**
378	**CPED1**	428	**PPP4C**	478	**REST**	528	**HS3ST3B1**	578	**TEP1**	628	**ECE2**	678	**GUK1**
379	**CRIM1**	429	**UQCRBP1**	479	**ITGA8**	529	**NBEAL1**	579	**HSPG2**	629	**EBF2**	679	**HNRNPA2B1**
380	**SNRPB**	430	**IKZF4**	480	**RAD54L2**	530	**NME2P1**	580	**PARP1**	630	**PDPR**	680	**KMT2C**

381	**MECP2**	431	**LINC00467**	481	**ERN 1.00**	531	**ATE1**	581	**FARSB**	631	**ATP5MG**	681	**INO80**
382	**RPS7**	432	**RPP40**	482	**TNS3**	532	**SSH1**	582	**IDS**	632	**TECPR2**	682	**RGPD6**
383	**RUVBL1**	433	**TGFBR2**	483	**RPA3**	533	**MYO18A**	583	**EIF3M**	633	**PIK3CG**	683	**CMC1**
384	**STARD8**	434	**RBX1**	484	**PROB1**	534	**H6PD**	584	**ROCK2**	634	**APEX1**	684	**CPSF3**
385	**PEAK1**	435	**APTX**	485	**EIF5A**	535	**BIRC6**	585	**UBE2I**	635	**ATP5MC3**	685	**GMNN**
386	**PTMA**	436	**PSMG3**	486	**MAGOH**	536	**SYNE2**	586	**RBM8A**	636	**CDCA3**	686	**TTC13**
387	**MYSM1**	437	**NUDT1**	487	**UCHL5**	537	**HCFC2**	587	**CHM**	637	**MCTS1**	687	**TAF1L**
388	**MRPL14**	438	**ITSN2**	488	**GARRE1**	538	**VPS45**	588	**CNPY2**	638	**FAM120A2P**	688	**ZXDA**
389	**CCNB1**	439	**ANKRD44**	489	**PSMB3**	539	**POLR1H**	589	**BAZ2B**	639	**ATP23**	689	**PCDHGA9**
390	**MIDEAS**	440	**NDUFB5**	490	**NDUFB2**	540	**RBL2**	590	**ACP1**	640	**NCOR1**	690	**ZNF660**
391	**PYGO1**	441	**ARHGEF17**	491	**ATP5MC2**	541	**AMIGO1**	591	**SFMBT2**	641	**UQCR10**	691	**IGSF9**
392	**ZNF641**	442	**KRBA2**	492	**PSMB5**	542	**CSF1**	592	**VRK1**	642	**GBAP1**	692	**BCCIP**
393	**SUMO1P3**	443	**NR3C1**	493	**PIAS1**	543	**PLSCR4**	593	**FKBPL**	643	**CCNT1**	693	**PA2G4P4**
394	**ENTREP3**	444	**DOCK1**	494	**FAM104B**	544	**TMA7**	594	**FAM72A**	644	**LRCH1**	694	**ADD1**
395	**SNRPA1**	445	**HNRNPL**	495	**CCPG1**	545	**RAN**	595	**NIFK**	645	**PDE3A**	695	**AFF1**
396	**FMN1**	446	**SNX30**	496	**PPOX**	546	**CALM2**	596	**MRPL11**	646	**MAN2A2**	696	**WASF2**
397	**HSD17B10**	447	**SFT2D1**	497	**ZNF827**	547	**PYGO2**	597	**CDKL5**	647	**TRAIP**	697	**USP12**
398	**TLN1**	448	**C19ORF48**	498	**LAMTOR5**	548	**FMNL3**	598	**NFAT5**	648	**ZNF32**	698	**RUSC1**
399	**RPS20**	449	**ANKFY1**	499	**TIMM8B**	549	**KAT2B**	599	**TACC1**	649	**PDCD2L**	699	**BUD23**
400	**MFN2**	450	**PIGU**	500	**CDK4**	550	**EXO1**	600	**ABT1**	650	**MFSD2B**	700	**NACA**

List 1 (cBioPortal)

	Gene		Gene		Gene		Gene		Gene		Gene
701	**AKAP11**	751	**SNORD116-28**	801	**ATP10D**	851	**PEX11B**	901	**PMVK**	951	**MRPS26**
702	**TAF11**	752	**ZNF70**	802	**TLCD1**	852	**CLTA**	902	**SLC8A1**	952	**HAUS8**
703	**RPL39L**	753	**RPL32**	803	**TRPM7**	853	**MRPL21**	903	**TUBA1C**	953	**ANP32B**
704	**TAF10**	754	**SIK3**	804	**ANAPC11**	854	**COA8**	904	**EPB41L2**	954	**COX5B**
705	**MRPL32**	755	**RBM43**	805	**ROR1**	855	**TMEM273**	905	**COQ3**	955	**MACF1**
706	**RPS10**	756	**SLC24A5**	806	**SVEP1**	856	**CHCHD2**	906	**HMCES**	956	**SRP19**
707	**NOL7**	757	**GIT2**	807	**RSC1A1**	857	**ADGRF5**	907	**MRPL27**	957	**NUP37**
708	**POLR2I**	758	**GPATCH2L**	808	**TRAP1**	858	**SPC25**	908	**SOD1**	958	**RPS19**
709	**ZKSCAN8**	759	**GOLT1A**	809	**PAFAH1B1**	859	**SEPTIN10**	909	**SYNRG**	959	**SCN7A**
710	**PLA2R1**	760	**OPHN1**	810	**AP4E1**	860	**HMGN1**	910	**TMEM14C**	960	**ITGB3BP**
711	**CMSS1**	761	**COMMD7**	811	**CDC123**	861	**PMM2**	911	**ZBTB20**	961	**ARHGEF39**
712	**NSL1**	762	**PAFAH1B3**	812	**CYCS**	862	**TXNL1**	912	**DOCK11**	962	**PRDM5**
713	**AKAP12**	763	**FEM1B**	813	**FYCO1**	863	**ADAM10**	913	**MALSU1**	963	**EPG5**
714	**CD93**	764	**TMEM127**	814	**OST4**	864	**DNAJC27**	914	**RIDA**	964	**ZNF592**
715	**ZNF609**	765	**KIF22**	815	**PTRHD1**	865	**SUV39H1**	915	**GBF1**	965	**PROX1**
716	**FAM72B**	766	**FOSL2**	816	**C19ORF53**	866	**OCIAD2**	916	**C12ORF73**	966	**TAF5L**
717	**AURKB**	767	**CHEK2**	817	**AHSA1**	867	**UNC5C**	917	**CDK1**	967	**ATP7A**
718	**NDUFAF2**	768	**MARCKSL1**	818	**DTYMK**	868	**N4BP1**	918	**P2RY1**	968	**BEND3P3**
719	**NEDD8**	769	**BTAF1**	819	**PTPRM**	869	**RPS18**	919	**BTBD8**	969	**NDUFB10**
720	**DENND5B**	770	**POP4**	820	**BYSL**	870	**ZC3HC1**	920	**PLEKHA7**	970	**ROMO1**
721	**PCBD1**	771	**TOP1P2**	821	**ACER2**	871	**CPEB4**	921	**NDUFV3**	971	**RAI1**

722	**TOMM22**	772	**MED13L**	822	**DPM3**	872	**ZC3H7B**	922	**NDUFA7**	972	**SON**
723	**MRPL52**	773	**DYNLRB1**	823	**THUMPD2**	873	**TEX30**	923	**DAZAP1**	973	**NCOR2**
724	**SBF2**	774	**PCNA**	824	**ZNF791**	874	**TEDC2**	924	**PABPN1**	974	**GIGYF2**
725	**ZFYVE26**	775	**TRIM44**	825	**TUBA1B**	875	**TRIR**	925	**MRPS15**	975	**PDCD2**
726	**ATP5ME**	776	**ARF5**	826	**NOP10**	876	**NR1I3**	926	**LSM 7.00**	976	**SMAD9**
727	**EYA3**	777	**RNF181**	827	**DIP2C**	877	**MRPL23**	927	**PCSK5**	977	**MRGBP**
728	**MEX3A**	778	**TAOK1**	828	**FAM161B**	878	**TPM3**	928	**GNS**	978	**C1QBP**
729	**MLF 2.00**	779	**INPP4A**	829	**NUTF2**	879	**LIFR**	929	**ZNF483**	979	**DPM2**
730	**HRH2**	780	**EEF1E1**	830	**NOTCH2**	880	**SNTB2**	930	**HMBS**	980	**TMEM177**
731	**MEA1**	781	**AGO1**	831	**RERE**	881	**NECTIN3**	931	**MCTP1**	981	**IL17RA**
732	**NOL10**	782	**NPM1**	832	**RBMS3**	882	**ZNF585B**	932	**POLD2**	982	**BACE1**
733	**THSD7A**	783	**NDUFAF4**	833	**NAV3**	883	**DDX39A**	933	**SNRNP25**	983	**CRY2**
734	**UTRN**	784	**NEK9**	834	**SH3D19**	884	**OXSM**	934	**SUSD1**	984	**ABHD2**
735	**NCOA2**	785	**IL6ST**	835	**SKA3**	885	**UBE2Q2P1**	935	**TCF12**	985	**MRPS5**
736	**PTPRB**	786	**RPL37A**	836	**SCAPER**	886	**GNAL**	936	**TIMM50**	986	**GPRASP1**
737	**PCDHGA12**	787	**SYNJ1**	837	**TEAD1**	887	**ITPA**	937	**VPS13B**	987	**CCT6A**
738	**DST**	788	**ARHGAP20**	838	**MTOR**	888	**FRY**	938	**MRPS12**	988	**LATS2**
739	**ARHGAP31**	789	**LRIG2**	839	**XYLT1**	889	**PTAFR**	939	**RPS6KA2**	989	**PHLPP2**
740	**PRDM11**	790	**KLF7**	840	**DDX59**	890	**SPECC1L**	940	**DLC1**	990	**ARPC3**
741	**ROCK1**	791	**RPS15A**	841	**PRKAR2A**	891	**MAN1A2**	941	**GAS7**	991	**PFN1**
742	**TSFM**	792	**PPA1**	842	**COX7A2L**	892	**RASEF**	942	**BIRC5**	992	**RO60**
743	**RBMX2**	793	**ADIPOR1**	843	**FBXO22-AS1**	893	**TJP1**	943	**EXOSC7**	993	**RPS16**
744	**EEF2KMT**	794	**CTSO**	844	**RPS29**	894	**GAB1**	944	**TRANK1**	994	**LAMA2**
745	**COA4**	795	**FRMD3**	845	**XCR1**	895	**COPS6**	945	**FLG**	995	**RFXANK**
746	**STMN1**	796	**METTL26**	846	**CMC2**	896	**POLR2G**	946	**DENND2C**	996	**RANBP1**
747	**TIMM8A**	797	**ATP5F1EP2**	847	**PDPK1**	897	**SIK2**	947	**CLIP1**	997	**NELFE**
748	**DAAM2**	798	**TTC28**	848	**EHD3**	898	**PSEN2**	948	**NFIC**	998	**ADAT1**
749	**SUMO2**	799	**ZNF154**	849	**COX17**	899	**DENND5A**	949	**RAE1**	999	**SETBP1**
750	**FNIP2**	800	**RAB11FIP5**	850	**ZEB1**	900	**PIK3C2A**	950	**FOXN3**	1000	**ITPR1**

B

List 2 (GEPIA)

	Gene		Gene		Gene		Gene		Gene		Gene
1	**SNRPEP2**	51	**STX6**	101	**C8orf59**	151	**LSM3**	201	**POMP**	251	**TMEM206**
2	**SNRPEP4**	52	**KIF14**	102	**GOLT1A**	152	**NCAPG**	202	**ARPC5**	252	**NDUFAB1**
3	**TMEM183A**	53	**SSR2**	103	**ZNF695**	153	**ZC3H11A**	203	**RP11-480I12.5**	253	**NUP37**
4	**TIMM17A**	54	**TADA1**	104	**NME7**	154	**MAGOHB**	204	**RFC4**	254	**MORN2**
5	**RFWD2**	55	**ASPM**	105	**PSMB4**	155	**CACYBPP2**	205	**DPY30**	255	**DROSHA**
6	**NUCKS1**	56	**GNPAT**	106	**SOX13**	156	**LINC00467**	206	**SRSF7**	256	**PEX11B**
7	**IPO9**	57	**TRMT10C**	107	**PPP1R15B**	157	**EEF1E1**	207	**MTERF3**	257	**EFNA4**
8	**TMEM81**	58	**FLAD1**	108	**CCNB1**	158	**TAF5L**	208	**TOR3A**	258	**DYNLT1**
9	**UBE2T**	59	**SNRPG**	109	**ADIPOR1**	159	**TOMM5**	209	**OR4M1**	259	**HNRNPU**
10	**RABIF**	60	**CKS1B**	110	**POLR2H**	160	**SSB**	210	**B4GALT3**	260	**FAM72D**
11	**ACBD6**	61	**C1orf112**	111	**VRK1**	161	**KIF15**	211	**TCEB1**	261	**SPRTN**

12	**EPRS**	62	**SSBP1**	112	**GGPS1**	162	**RP11-242C24.3**	212	**PARPBP**	262	**NAE1**
13	**GLRX2**	63	**WDR12**	113	**CEBPZ**	163	**KIAA0020**	213	**SNAP47**	263	**KIF18A**
14	**CCT3**	64	**MRPL3**	114	**KRTCAP2**	164	**OR7D4**	214	**FAM189B**	264	**DLGAP5**
15	**LYPLAL1**	65	**NOP58**	115	**MGST3**	165	**TBC1D7**	215	**CPSF3**	265	**PALB2**
16	**CNIH4**	66	**USMG5**	116	**USP21**	166	**URB2**	216	**CHEK2**	266	**HDAC2**
17	**CCDC58**	67	**MRPL24**	117	**PSMD4**	167	**UMPS**	217	**PTTG1**	267	**RIOK2**
18	**RBBP5**	68	**ARL8A**	118	**TAF11**	168	**RAN**	218	**ARF1**	268	**CKS2**
19	**COA6**	69	**LIN9**	119	**RP11-651P23.4**	169	**ENY2**	219	**UBAP2L**	269	**SGOL2**
20	**DAP3**	70	**TMEM9**	120	**RP11-10N23.4**	170	**MCTS1**	220	**ADSS**	270	**GTF3C6**
21	**PRDX6**	71	**PIGC**	121	**SPC25**	171	**RPP30**	221	**PPIL1**	271	**HDDC2**
22	**DHX9**	72	**CENPF**	122	**PDSS1**	172	**POGK**	222	**GMNN**	272	**RP11-480I12.7**
23	**H3F3A**	73	**FLVCR1**	123	**RP11-345J4.5**	173	**MRPL51**	223	**RFC2**	273	**NOL7**
24	**DDX59**	74	**NVL**	124	**TRIAP1**	174	**RUVBL1**	224	**CCT8**	274	**CSNK2B**
25	**MRPL9**	75	**BRIX1**	125	**SNRPD1**	175	**PFDN2**	225	**LSM5**	275	**PSMA1**
26	**RBM34**	76	**TPR**	126	**SMYD2**	176	**ATIC**	226	**CHTOP**	276	**MRPL55**
27	**KLHL12**	77	**UFC1**	127	**MEX3A**	177	**CENPH**	227	**PMF1**	277	**TAF5**
28	**INTS7**	78	**TPRKB**	128	**TOMM20**	178	**GEMIN6**	228	**AL513523.2**	278	**PSMA3**
29	**FH**	79	**PARP1**	129	**H2AFZ**	179	**DDX10**	229	**KIF20A**	279	**POLE2**
30	**METTL13**	80	**PSMA2**	130	**KIAA1614-AS1**	180	**YOD1**	230	**COX20**	280	**TCP1**
31	**PRCC**	81	**MTX1**	131	**RP11-452F19.3**	181	**PIGM**	231	**ZNF124**	281	**BPNT1**
32	**TBCE**	82	**SCAMP3**	132	**MTFR2**	182	**JMJD4**	232	**PPIH**	282	**SNRPGP10**
33	**CACYBP**	83	**RAB25**	133	**SDHC**	183	**JTB**	233	**RP11-295G20.2**	283	**HNRNPA3**
34	**TSEN15**	84	**METTL18**	134	**GAR1**	184	**GINS1**	234	**SUV39H2**	284	**CDC45**
35	**TFB2M**	85	**NDUFAF4**	135	**DIEXF**	185	**KB-1568E2.1**	235	**MTBP**	285	**BROX**
36	**C1orf131**	86	**CENPL**	136	**TIMM8A**	186	**GTF2H5**	236	**LAMTOR2**	286	**SNRPGP2**
37	**EXO1**	87	**MRPL1**	137	**NUP205**	187	**TSACC**	237	**COQ3**	287	**NCAPH**
38	**MRPL13**	88	**DSTYK**	138	**LRPPRC**	188	**IARS2**	238	**SKA3**	288	**MSTO1**
39	**NEK2**	89	**RRP15**	139	**UBQLN4**	189	**AHCTF1**	239	**RMI2**	289	**BTF3L4**
40	**TIPRL**	90	**CDC73**	140	**RBM8A**	190	**C1orf43**	240	**SARNP**	290	**LINC00824**
41	**DNAH14**	91	**SRP9**	141	**DUSP12**	191	**SUMO1**	241	**GPR89B**	291	**VPS45**
42	**NUP133**	92	**KDM5B**	142	**TMEM14B**	192	**ESRP1**	242	**WDHD1**	292	**SLC50A1**
43	**HAX1**	93	**ILF2**	143	**TTK**	193	**THOC7**	243	**YARS2**	293	**COX20P1**
44	**UCK2**	94	**SF3B4**	144	**SNAPIN**	194	**RAD54B**	244	**ABRACL**	294	**KIF11**
45	**RP11-101E13.5**	95	**NTPCR**	145	**RPF2**	195	**ARV1**	245	**KIN**	295	**DSCC1**
46	**UCHL5**	96	**METTL5**	146	**FARSB**	196	**APOA1BP**	246	**CCT4**	296	**COX7A2**
47	**MRPS14**	97	**EIF2D**	147	**VPS72**	197	**ATP5C1**	247	**HDGF**	297	**MIS18A**
48	**FAM72A**	98	**DARS2**	148	**RPP40**	198	**H2AFV**	248	**TBPL1**	298	**RPP38**
49	**LSM2**	99	**NUF2**	149	**PHF14**	199	**GCFC2**	249	**CDKN3**	299	**ERCC6L**
50	**SNRPC**	100	**SCNM1**	150	**NDUFS2**	200	**NOL10**	250	**FBXO28**	300	**MTCH2**

List 2 (GEPIA)

	Gene		Gene		Gene		Gene		Gene		Gene
301	**SFT2D1**	351	**CDCA3**	401	**TEX30**	451	**RP11-435O5.2**	501	**CMC1**	551	**ADCK3**
302	**CWC27**	352	**DTL**	402	**MAD2L1**	452	**BPHL**	502	**HOXC13**	552	**LINC00493**

303	**GMPS**	353	**LMNB1**	403	**RPS27**	453	**PCNA**	503	**PXN-AS1**	553	**PSMB5**
304	**TTC13**	354	**KNTC1**	404	**SMG7**	454	**CENPE**	504	**AURKB**	554	**CCNB2**
305	**C12orf45**	355	**DCUN1D5**	405	**CBX3**	455	**EIF3E**	505	**SFXN4**	555	**USF1**
306	**CTD-2124B8.2**	356	**MRPL15**	406	**KIF4A**	456	**HNRNPL**	506	**RP4-616B8.5**	556	**CHD1L**
307	**PCAT6**	357	**NDUFB3**	407	**MRPS9**	457	**HSPE1**	507	**BLZF1**	557	**RPL39L**
308	**PRUNE**	358	**NDUFA12**	408	**CCNA2**	458	**PNPT1**	508	**OIP5**	558	**NUTF2**
309	**CHEK1**	359	**SNRPF**	409	**PDCD2L**	459	**TBCA**	509	**TRIT1**	559	**NONO**
310	**ITGB3BP**	360	**HELLS**	410	**ZNF672**	460	**TXN**	510	**TBP**	560	**C20orf24**
311	**INTS8**	361	**RNF187**	411	**LRR1**	461	**RP11-386G11.10**	511	**KIF2C**	561	**PSME4**
312	**TIMM23**	362	**MRPS33**	412	**DEDD**	462	**TRAP1**	512	**TPM3**	562	**AC006547.8**
313	**EIF2S2**	363	**MRPL42**	413	**SS18L2**	463	**FBXO5**	513	**LYSMD1**	563	**MCM6**
314	**RP11-298D21.1**	364	**SGOL1**	414	**LTV1**	464	**PRIM2**	514	**EBNA1BP2**	564	**LYRM4**
315	**RNPEP**	365	**RIOK1**	415	**RANBP1**	465	**NIT2**	515	**REPS1**	565	**CDK8**
316	**DNAJC2**	366	**TOR1AIP2**	416	**BOLA3**	466	**SPC24**	516	**TMEM261**	566	**GGCT**
317	**USP39**	367	**DCTPP1**	417	**ACTL6A**	467	**TIMM9**	517	**FAM72B**	567	**NCBP2**
318	**PSMB1**	368	**RPS6KC1**	418	**PTGES3**	468	**MELK**	518	**EBPL**	568	**GEMIN2**
319	**PDCL3**	369	**DCAF6**	419	**C12orf73**	469	**PSMG2**	519	**NUDCD1**	569	**HMMR**
320	**WDR75**	370	**C1orf35**	420	**PSMD1**	470	**DSN1**	520	**RRM1**	570	**E2F5**
321	**CCSAP**	371	**IGHV2OR16-5**	421	**TATDN3**	471	**DEPDC1B**	521	**RNASEH2A**	571	**SENP3**
322	**NSL1**	372	**RP11-343C2.12**	422	**MEMO1**	472	**ALKBH2**	522	**ZNF670**	572	**CHML**
323	**NDUFS5**	373	**EHMT2**	423	**ECT2**	473	**ZC3H8**	523	**LINC01136**	573	**RPL30**
324	**SF3B6**	374	**C1orf27**	424	**PRRC2C**	474	**SAYSD1**	524	**PDCD2**	574	**NEIL3**
325	**IGSF9**	375	**STMN1**	425	**FKBPL**	475	**PTRHD1**	525	**HSPA4**	575	**SPDL1**
326	**GPATCH4**	376	**NDC80**	426	**NOL11**	476	**ACP1**	526	**C18orf21**	576	**DRG1**
327	**FAM104B**	377	**BUB1B**	427	**ACOT13**	477	**RPP21**	527	**HIST1H3PS1**	577	**ALG6**
328	**DYNLL1**	378	**ISG20L2**	428	**PWP1**	478	**DNA2**	528	**CEP55**	578	**CDC123**
329	**RAB4A**	379	**KIAA0101**	429	**HYLS1**	479	**TOMM40L**	529	**TAF1A**	579	**SUCO**
330	**NUP155**	380	**UAP1**	430	**MRPS21**	480	**CDC20**	530	**GLRX5**	580	**C1QBP**
331	**ORC3**	381	**RPA3**	431	**EIF5B**	481	**ZNF669**	531	**MCM3**	581	**KB-1208A12.3**
332	**CMSS1**	382	**C4orf27**	432	**FAM20B**	482	**C2orf76**	532	**APH1A**	582	**RP3-337H4.9**
333	**KIFAP3**	383	**HEATR1**	433	**CDK1**	483	**C14orf2**	533	**NUP85**	583	**TUBB**
334	**DESI2**	384	**RBM45**	434	**CCDC138**	484	**FANCD2**	534	**SKA1**	584	**DNAJB11**
335	**ASUN**	385	**GLMP**	435	**PLK4**	485	**DONSON**	535	**CAMSAP2**	585	**TXNL1**
336	**CCDC167**	386	**RP13-753N3.3**	436	**MRPL18**	486	**SPAG5**	536	**RAD54L**	586	**TARBP1**
337	**TMCO1**	387	**TRAIP**	437	**C8orf76**	487	**CTD-2369P2.12**	537	**NCSTN**	587	**WDYHV1**
338	**EMG1**	388	**HCG25**	438	**HPRT1**	488	**UTP6**	538	**HMGN3**	588	**SLC25A44**
339	**BTF3L4P2**	389	**ERH**	439	**USP2-AS1**	489	**RP11-132A1.4**	539	**EIF2B3**	589	**CHAC2**
340	**PDCD5**	390	**HNRNPAB**	440	**MCUR1**	490	**SAAL1**	540	**CALM2**	590	**UQCRH**
341	**ANAPC7**	391	**SH3BP5L**	441	**TROAP**	491	**CDC25A**	541	**ELAVL1**	591	**RFESD**
342	**PAICS**	392	**CNOT10**	442	**ANP32E**	492	**MPHOSPH10**	542	**RP11-344N17.8**	592	**SNX27**
343	**NOP56**	393	**MLF1**	443	**PAK1IP1**	493	**PPIA**	543	**PIK3R4**	593	**KATNA1**
344	**MRPL47**	394	**EZH2**	444	**CCZ1**	494	**NENF**	544	**MDM4**	594	**KIF23**
345	**DCAF13**	395	**RPL26L1**	445	**RPL22L1**	495	**NIFK**	545	**IQGAP3**	595	**TAGLN2**
346	**UBE2C**	396	**AIDA**	446	**TXNDC17**	496	**CDC25C**	546	**UHRF1**	596	**BUB1**

347	**TPX2**	397	**PSMG1**	447	**LYAR**	497	**WDR70**	547	**POLR1C**	597	**MND1**
348	**CCDC59**	398	**MAGOH**	448	**CPSF6**	498	**NUP35**	548	**ABT1**	598	**GAS5**
349	**SETDB1**	399	**ZSCAN16**	449	**PSMD14**	499	**TROVE2**	549	**MORC2**	599	**CMC2**
350	**ELK4**	400	**FAM83D**	450	**PRMT5**	500	**POLR3F**	550	OXSM	600	**ABCB10**

List 2 (GEPIA)

	Gene		Gene		Gene		Gene		Gene		Gene
601	**CENPN**	651	**SLC41A1**	701	**THUMPD2**	751	**YY1AP1**	801	**DNAJC9**	851	**C5orf34**
602	**CENPQ**	652	**PLK1**	702	**SMC4**	752	**ATAD5**	802	**UCHL3**	852	**RP3-337H4.8**
603	**TTC27**	653	**ATP5J**	703	**GNL2**	753	**RP11-135L13.4**	803	**TOMM70A**	853	**NUP93**
604	**DGUOK**	654	**NKRF**	704	**C7orf73**	754	**STAMBP**	804	**THOC1**	854	**TLCD1**
605	**POC1A**	655	**GTPBP4**	705	**MDH1**	755	**PKMYT1**	805	**RP11-122A21.2**	855	**PFDN6**
606	**FANCG**	656	**RAD51AP1**	706	**ZBTB41**	756	**RPIA**	806	**CWC15**	856	**CLTA**
607	**GTF2H4**	657	**NUP88**	707	**CHCHD4**	757	**CDK2AP1**	807	**HRSP12**	857	**ANP32B**
608	**UBE2V2**	658	**KPNA2**	708	**TARS**	758	**TRIP13**	808	**ZNF706**	858	**POLR3G**
609	**TMEM258**	659	**RP11-332M2.1**	709	**FIGNL1**	759	**PPP1R14B**	809	**PARP2**	859	**E2F1**
610	**GLRX3**	660	**C11orf74**	710	**AC009005.2**	760	**CCDC77**	810	**POLQ**	860	**FAM111B**
611	**EPCAM**	661	**RP11-78A19.3**	711	**COG2**	761	**IMMP1L**	811	**S100A11**	861	**SNRPEP6**
612	**AIMP2**	662	**SNRPB2**	712	**PVT1**	762	**ACTR6**	812	**CTPS1**	862	**CRIPT**
613	**MARC1**	663	**MARCKSL1**	713	**TCAIM**	763	**GTF3C2**	813	**PRIM1**	863	**HMGN1**
614	**BLM**	664	**SOX4**	714	**RPUSD3**	764	**WRNIP1**	814	**NDUFB6**	864	**ZNRD1**
615	**ATP5O**	665	**MDN1**	715	**UQCRB**	765	**YWHAQ**	815	**ZC3HC1**	865	**WDR53**
616	**RP11-1100L3.7**	666	**SRPK1**	716	**LAD1**	766	**TRIM11**	816	**MRPL39**	866	**HSPA14**
617	**NUDT5**	667	**CENPI**	717	**TXNL4A**	767	**NDUFAF2**	817	**SASS6**	867	**COX5A**
618	**DUSP23**	668	**PTMA**	718	**CDCA7**	768	**ZNF93**	818	**FAM49B**	868	**UBL5**
619	**EXOSC9**	669	**SCML2**	719	**VDAC1**	769	**MTHFD1**	819	**MRPS22**	869	**RP11-480I12.9**
620	**POLR2K**	670	**RAB3GAP2**	720	**ZNF670**	770	**PBK**	820	**MLK4**	870	**TMEM177**
621	**KIFC1**	671	**AP000251.3**	721	**ALDH9A1**	771	**RP3-461F17.3**	821	**MSH2**	871	**TCERG1**
622	**CDCA8**	672	**HJURP**	722	**H3F3AP4**	772	**PDCD11**	822	**E2F3**	872	**RP11-23E19.2**
623	**DBF4**	673	**ORC5**	723	**IPO4**	773	**RP11-10G12.1**	823	**MRPL30**	873	**EXOSC8**
624	**BOLA1**	674	**FDPS**	724	**C17orf53**	774	**TMEM186**	824	**ERI3**	874	**EXOSC1**
625	**CTB-193M12.5**	675	**KLHDC9**	725	**GPR89A**	775	**ATP5J2**	825	**FEN1**	875	**RPL39**
626	**CENPK**	676	**MRS2**	726	**ASF1A**	776	**ANKRD32**	826	**CCNE2**	876	**HMGB2**
627	**MCM10**	677	**RP11-343H5.6**	727	**MASTL**	777	**RSL1D1**	827	**NUSAP1**	877	**NDUFB11**
628	**UBE2Q1**	678	**ZNF317**	728	**VASH2**	778	**ZUFSP**	828	**RANP1**	878	**PIGU**
629	**PVRL4**	679	**CECR5**	729	**DNTTIP2**	779	**CENPA**	829	**TOP2A**	879	**RP11-244M2.1**
630	**HNRNPC**	680	**SRSF1**	730	**VARS**	780	**COMMD3**	830	**ZNF32**	880	**NDC1**
631	**BZW2**	681	**RP11-395L14.17**	731	**RP11-97O12.5**	781	**CHCHD1**	831	**C11orf98**	881	**AUNIP**
632	**RP11-402J6.3**	682	**SEH1L**	732	**NDUFAF5**	782	**CTB-43P18.1**	832	**VANGL2**	882	**RP11-770J1.5**
633	**ARMC10**	683	**PNO1**	733	**COA5**	783	**CKAP2L**	833	**LRRC40**	883	**PMS1**
634	**POLR3C**	684	**HNRNPA2B1**	734	**SKP2**	784	**BUD31**	834	**LINC00665**	884	**COX7A2L**
635	**MRPS35**	685	**MRPL22**	735	**DEGS1**	785	**RFC3**	835	**TUBAP2**	885	**ZC3H15**
636	**FANCB**	686	**C11orf73**	736	**DNMT3B**	786	**TMSB15A**	836	**SMN1**	886	**PSMA5**
637	**DENR**	687	**ARHGEF39**	737	**GTF2IRD1**	787	**MRPS5**	837	**RACGAP1**	887	**RGS5**

638	**SLC25A3**	688	**SMARCA4**	738	**POLE**	788	**UQCC2**	838	**EIF2B1**	888	**COPS5**
639	**TBC1D31**	689	**HN1**	739	**SRP19**	789	**NASP**	839	**GEN1**	889	**AHSA1**
640	**PAICSP4**	690	**TSEN2**	740	**RHEB**	790	**TAF1D**	840	**XRCC4**	890	**FUS**
641	**UNG**	691	**SHFM1**	741	**MCM2**	791	**PUS7**	841	**APOO**	891	**CBWD2**
642	**MRPL19**	692	**TMEM97**	742	**LINC00998**	792	**PRPF3**	842	**VTA1**	892	**SNX14**
643	**RTKN2**	693	**TRIM27**	743	**RAB29**	793	**UTP11L**	843	**PUS1**	893	**TFDP2**
644	**HMGB1**	694	**NPM3**	744	**IAH1**	794	**MRPL17**	844	**CDK5RAP1**	894	**SYNJ2BP-COX16**
645	**TIMMDC1**	695	**NGDN**	745	**LAGE3**	795	**CREB3L4**	845	**RBM8B**	895	**PAIP1**
646	**COX16**	696	**EIF2A**	746	**U2SURP**	796	**RP11-667K14.4**	846	**XRCC6BP1**	896	**MTIF2**
647	**FBL**	697	**MRPS15**	747	**GLO1**	797	**SPATA5L1**	847	**SBK1**	897	**FOXP4-AS1**
648	**CDC7**	698	**RFC5**	748	**SERTAD4**	798	**ORC6**	848	**HSBP1**	898	**POLA2**
649	**SRSF2**	699	**SLBP**	749	**PYGO2**	799	**TATDN1**	849	**SYNCRIP**	899	**HCG17**
650	**MRPL14**	700	**RAD1**	750	**DHX57**	800	**HSP90AA1**	850	**CCZ1B**	900	**APEX1**

List 2 (GEPIA)

	Gene		Gene
901	**TXLNG**	951	**MCM7**
902	**UXT**	952	**C1orf106**
903	**TMEM70**	953	**GLMN**
904	**TXNDC9**	954	**PPP1CC**
905	**RP1-228H13.5**	955	**RPAP3**
906	**MGME1**	956	**MMACHC**
907	**APTX**	957	**MTA3**
908	**ZNF883**	958	**HAUS8**
909	**MRPL35**	959	**DNMT1**
910	**TRA2B**	960	**MRPL33**
911	**DHFRP1**	961	**ARMC1**
912	**SNHG1**	962	**FOXM1**
913	**CUL3**	963	**TMPO**
914	**GTPBP10**	964	**COMMD7**
915	**RBMX**	965	**HMCES**
916	**RHNO1**	966	**ACBD3**
917	**PPA2**	967	**PPP2R5A**
918	**FBXO45**	968	**EFTUD2**
919	**SNRPD2**	969	**ARPC1A**
920	**ZYG11A**	970	**PSMB2**
921	**CEP78**	971	**MRPS18C**
922	**RAD51**	972	**PIP5K1A**
923	**DHTKD1**	973	**FXN**
924	**C16orf59**	974	**ZNF638**
925	**GTF3A**	975	**GATAD2B**
926	**RP11-126K1.2**	976	**HSPD1**
927	**RP5-967N21.11**	977	**THOC3**
928	**BIRC5**	978	**DDX56**

929	**MTX2**	979	**SAP30**
930	**STRAP**	980	**H3F3AP6**
931	**CDCA5**	981	**SLC19A1**
932	**UBE3D**	982	**RP11-429G19.3**
933	**SLIRP**	983	**MAD2L1BP**
934	**PMVK**	984	**SF3B5**
935	**CSE1L**	985	**HSPH1**
936	**HOXC10**	986	**NDUFA9**
937	**POLR1B**	987	**CRLS1**
938	**GORAB**	988	**MRPS16**
939	**KIF20B**	989	**KIAA1524**
940	**OPN3**	990	**SUPT16H**
941	**HMGA1**	991	**DUT**
942	**XPO1**	992	**DNAJA1**
943	**TSNAX**	993	**METAP2**
944	**COCH**	994	**DTYMK**
945	**PCGF6**	995	**C14orf166**
946	**RRNAD1**	996	**HNRNPA3P5**
947	**ZNF281**	997	**TIAL1**
948	**LCLAT1**	998	**RP11-968A15.2**
949	**NRD1**	999	**PPOX**
950	**TUBA1B**	1000	**UBE2K**

C

345 genes intersected by list1 and list2

	Gene		Gene		Gene		Gene		Gene		Gene		Gene
1	**HMCES**	51	**MRPL47**	101	**MEMO1**	151	**MSTO1**	201	**MRPL3**	251	**PSMD4**	301	**GTF3C6**
2	**KRTCAP2**	52	**DYNLT1**	102	**SLC50A1**	152	**S100A11**	202	**POMP**	252	**JMJD4**	302	**TLCD1**
3	**DPY30**	53	**FAM72B**	103	**PPOX**	153	**SRP9**	203	**CDCA3**	253	**FLAD1**	303	**SF3B4**
4	**GOLT1A**	54	**EFNA4**	104	**CMC1**	154	**PTTG1**	204	**AHSA1**	254	**POLR2H**	304	**TPRKB**
5	**COX7A2L**	55	**COA6**	105	**ARHGEF39**	155	**RPP30**	205	**TFB2M**	255	**LIN9**	305	**NUDT5**
6	**PDCL3**	56	**NUP37**	106	**METTL5**	156	**PEX11B**	206	**TIMM9**	256	**USF1**	306	**MORN2**
7	**ARV1**	57	**CENPH**	107	**GTF2H5**	157	**SNAPIN**	207	**TBCE**	257	**RPA3**	307	**SNRPB2**
8	**COX5A**	58	**SF3B6**	108	**LRR1**	158	**STMN1**	208	**NOP56**	258	**TMEM14B**	308	**HDGF**
9	**TAF5L**	59	**IMMP1L**	109	**RUVBL1**	159	**RAN**	209	**HNRNPA2B1**	259	**ADIPOR1**	309	**PIGU**
10	**BRIX1**	60	**TSEN15**	110	**THOC7**	160	**UBE2T**	210	**PPP1R14B**	260	**THUMPD2**	310	**PSMB1**
11	**GAS5**	61	**COMMD7**	111	**RANBP1**	161	**UFC1**	211	**OXSM**	261	**DDX59**	311	**TXN**
12	**CCDC59**	62	**MTX1**	112	**CSNK2B**	162	**PDCD2**	212	**ZC3HC1**	262	**TIPRL**	312	**BOLA1**
13	**SUMO1**	63	**EBPL**	113	**NSL1**	163	**GAR1**	213	**NDUFAF2**	263	**SDHC**	313	**SFT2D1**
14	**TOMM20**	64	**LAGE3**	114	**CHCHD1**	164	**RPF2**	214	**UQCC2**	264	**RNASEH2A**	314	**TRMT10C**
15	**DNAH14**	65	**COX7A2**	115	**RNF187**	165	**CKS2**	215	**TBCA**	265	**NDUFB11**	315	**SFXN4**
16	**POLR1C**	66	**ARF1**	116	**SLIRP**	166	**HNRNPC**	216	**UBE2C**	266	**B4GALT3**	316	**TEX30**
17	**CCNB1**	67	**SNRPF**	117	**TRAIP**	167	**MRPL22**	217	**MEX3A**	267	**TBC1D7**	317	**RRP15**
18	**PTRHD1**	68	**EMG1**	118	**TXNL1**	168	**ITGB3BP**	218	**MRPL13**	268	**NOL7**	318	**STX6**

19	**NDUFAF4**	69	**MRPL15**	119	**MRPS9**	169	**TIMM8A**	219	**CMC2**	269	**NVL**	319	**PIGC**
20	**NDUFAB1**	70	**PSMG2**	120	**PDCD2L**	170	**NDUFB3**	220	**USP21**	270	**DTL**	320	**SSBP1**
21	**IPO9**	71	**PUS1**	121	**RPL39**	171	**PARP1**	221	**MRPL55**	271	**TOMM5**	321	**TXNDC17**
22	**MRPL1**	72	**NOP58**	122	**APTX**	172	**CHTOP**	222	**ENY2**	272	**CBX3**	322	**ANP32B**
23	**HMGN1**	73	**RPL22L1**	123	**SNRPG**	173	**DUSP23**	223	**MRPS5**	273	**CDC123**	323	**HNRNPL**
24	**MRPS14**	74	**TUBA1B**	124	**COQ3**	174	**CCDC167**	224	**APH1A**	274	**ACP1**	324	**HNRNPU**
25	**PRDX6**	75	**ZNF672**	125	**TMEM183A**	175	**PSMA2**	225	**MRPL14**	275	**TATDN3**	325	**CDKN3**
26	**ILF2**	76	**MAGOHB**	126	**PYGO2**	176	**FH**	226	**DTYMK**	276	**MARCKSL1**	326	**UBE2Q1**
27	**AIDA**	77	**AURKB**	127	**PPIA**	177	**ZNF695**	227	**PFDN6**	277	**TSACC**	327	**PRCC**
28	**PSMA3**	78	**CKS1B**	128	**GEMIN6**	178	**SKA3**	228	**TRIM11**	278	**ZNF32**	328	**RPL26L1**
29	**FARSB**	79	**ARPC5**	129	**NTPCR**	179	**MCTS1**	229	**EEF1E1**	279	**NIT2**	329	**FDPS**
30	**CLTA**	80	**ANAPC7**	130	**NDUFB6**	180	**HDDC2**	230	**DYNLL1**	280	**MRPS15**	330	**RMI2**
31	**PDCD5**	81	**NDUFS2**	131	**TXNL4A**	181	**DUSP12**	231	**UBL5**	281	**CACYBP**	331	**NOL10**
32	**DGUOK**	82	**NENF**	132	**ERH**	182	**PTMA**	232	**HNRNPAB**	282	**MRPS21**	332	**SCNM1**
33	**RPP40**	83	**SCAMP3**	133	**RAB25**	183	**NUTF2**	233	**RAB4A**	283	**LINC00467**	333	**VRK1**
34	**BIRC5**	84	**TTC13**	134	**MRPL17**	184	**COX16**	234	**APOO**	284	**RBM8A**	334	**NEK2**
35	**TAGLN2**	85	**SS18L2**	135	**ABRACL**	185	**MRPL18**	235	**PSMB4**	285	**MAGOH**	335	**TRAP1**
36	**DAP3**	86	**UMPS**	136	**TRIAP1**	186	**METTL18**	236	**TAF11**	286	**EXO1**	336	**VPS45**
37	**MIS18A**	87	**C1QBP**	137	**UXT**	187	**RABIF**	237	**CCT3**	287	**TMEM258**	337	**UQCRH**
38	**ALKBH2**	88	**TIMM17A**	138	**APEX1**	188	**NUCKS1**	238	**HSPE1**	288	**NDUFA12**	338	**LYPLAL1**
39	**LAMTOR2**	89	**IGSF9**	139	**SF3B5**	189	**RBM34**	239	**TMEM177**	289	**NDUFS5**	339	**RPP38**
40	**BUD31**	90	**MRPS33**	140	**RFC4**	190	**DEDD**	240	**PSMB5**	290	**RPP21**	340	**TMEM81**
41	**UBQLN4**	91	**PDSS1**	141	**RPL30**	191	**TMEM9**	241	**SSR2**	291	**VPS72**	341	**PFDN2**
42	**GLRX2**	92	**CPSF3**	142	**HAUS8**	192	**MTFR2**	242	**SPC25**	292	**GNPAT**	342	**UCK2**
43	**MRPL24**	93	**FKBPL**	143	**SRP19**	193	**PPIH**	243	**CHEK2**	293	**GMNN**	343	**EIF2D**
44	**TPM3**	94	**CENPL**	144	**TADA1**	194	**RFC2**	244	**RNPEP**	294	**NDUFA9**	344	**RPL39L**
45	**MGST3**	95	**GGPS1**	145	**MRPL9**	195	**BPNT1**	245	**TMCO1**	295	**SARNP**	345	**SNRPD2**
46	**CDK1**	96	**PMVK**	146	**ABT1**	196	**NIFK**	246	**MRPS16**	296	**FAM104B**		
47	**ACBD6**	97	**SNRPC**	147	**NUF2**	197	**CMSS1**	247	**FAM72A**	297	**UCHL5**		
48	**JTB**	98	**SNRPD1**	148	**CALM2**	198	**EXOSC1**	248	**FBL**	298	**BOLA3**		
49	**TOR3A**	99	**UQCRB**	149	**EXOSC8**	199	**SRSF7**	249	**CNIH4**	299	**MRPL51**		
50	**PMF1**	100	**PCNA**	150	**ARL8A**	200	**SH3BP5L**	250	**GGCT**	300	**HAX1**		

Supplementary Figure 2. The lists of genes used for pan-cancer enrichment analysis. (A) list1: The 1000 SNRPE correlated genes obtained through the cBioPortal tool. (B) list2: The top 1000 1000 SNRPE correlated genes obtained through the GEPIA tool. (C) The 345 genes intersected by list1 and list2.

Supplementary Figure 3. Functional enrichment analysis of SNRPE in breast cancer.

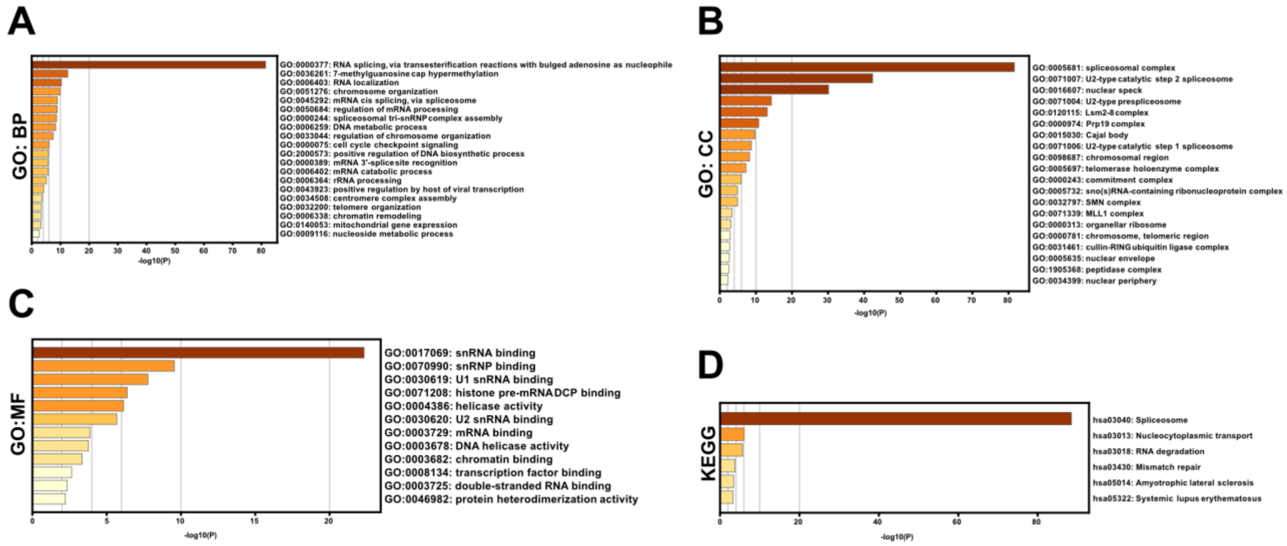

Supplementary Figure 3. Functional enrichment analysis of SNRPE in breast cancer. (A-C) GO enrichment analysis of the 345 SNRPE correlated genes in breast cancer. (D) KEGG enrichment analysis of the 345 SNRPE correlated genes in breast cancer.
